# Metabolic Imaging Detects Resistance to PI3Kα Inhibition Mediated by Persistent FOXM1 Expression in ER^+^ Breast Cancer

**DOI:** 10.1016/j.ccell.2020.08.016

**Published:** 2020-10-12

**Authors:** Susana Ros, Alan J. Wright, Paula D'Santos, De-en Hu, Richard L. Hesketh, Yaniv Lubling, Dimitra Georgopoulou, Giulia Lerda, Dominique-Laurent Couturier, Pedram Razavi, Rapahel Pelossof, Ankita S. Batra, Elizabeth Mannion, David Y. Lewis, Alistair Martin, Richard D. Baird, Mafalda Oliveira, Leonora W. de Boo, Sabine C. Linn, Maurizio Scaltriti, Oscar M. Rueda, Alejandra Bruna, Carlos Caldas, Kevin M. Brindle

**Affiliations:** 1 Cancer Research UK Cambridge Institute and Department of Oncology, Li Ka Shing Centre, University of Cambridge, Cambridge, UK; 2 Cancer Research UK Cambridge Cancer Centre, Cambridge, UK; 3 Human Oncology and Pathogenesis Program, X and Department of Pathology, Y and Department of Medicine, Memorial Sloan Kettering Cancer Center, New York, NY, USA; 4 Breast Cancer Research Programme, Cancer Research UK Cambridge Centre, Cambridge, UK; 5 Medical Oncology, Vall d’Hebron Hospital, Vall d’Hebron Institute of Oncology (VHIO), Barcelona, Spain; 6 Division of Molecular Pathology, The Netherlands Cancer Institute, Amsterdam, the Netherlands; 7 Department of Pathology, University Medical Center Utrecht, Utrecht, the Netherlands; 8 Department of Biochemistry, University of Cambridge, Cambridge UK

**Keywords:** breast cancer, PI3K alpha inhibition, lactate dehydrogenase, FOXM1, hexokinase 2, MRI, hyperpolarized [1-^13^C]pyruvate, FDG-PET, treatment response, biomarker

## Abstract

*PIK3CA*, encoding the PI3Kα isoform, is the most frequently mutated oncogene in estrogen receptor (ER)-positive breast cancer. Isoform-selective PI3K inhibitors are used clinically but intrinsic and acquired resistance limits their utility. Improved selection of patients that will benefit from these drugs requires predictive biomarkers. We show here that persistent FOXM1 expression following drug treatment is a biomarker of resistance to PI3Kα inhibition in ER^+^ breast cancer. FOXM1 drives expression of lactate dehydrogenase (LDH) but not hexokinase 2 (HK-II). The downstream metabolic changes can therefore be detected using MRI of LDH-catalyzed hyperpolarized ^13^C label exchange between pyruvate and lactate but not by positron emission tomography measurements of HK-II-mediated trapping of the glucose analog 2-deoxy-2-[^18^F]fluorodeoxyglucose. Rapid assessment of treatment response in breast cancer using this imaging method could help identify patients that benefit from PI3Kα inhibition and design drug combinations to counteract the emergence of resistance.

## Highlights

• Imaging hyperpolarized [1-^13^C]pyruvate metabolism detects response to PI3Kα inhibitors
• PI3Kα inhibitors downregulate FOXM1 expression in ER^+^ breast tumor models
• FOXM1 induces LDH expression and ^13^C label exchange between pyruvate and lactate
• Imaging [1-^13^C]pyruvate metabolism could be used clinically to guide treatment

## Graphical Abstract

**Figure:**
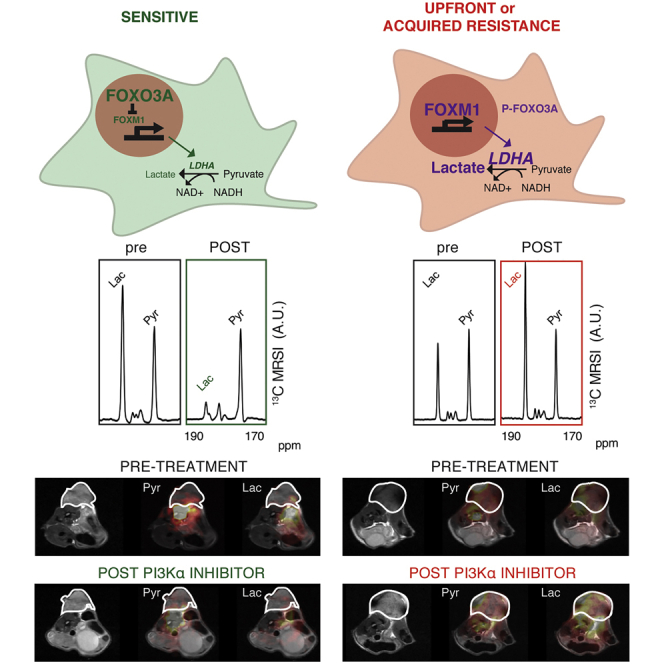

## Teaser

Ros et al. find that imaging of hyperpolarized [1-^13^C]pyruvate metabolism allows early detection of response and resistance to PI3Kα inhibition in *PIK3CA*-mutant ER^+^ breast cancer, which could be used clinically to guide treatment. Increased expression of FOXM1 results in resistance to PI3Kα inhibition and sustains LDH expression.

## Introduction

PIK3CA encodes the p110α subunit of the class 1 phosphoinositide 3-kinase (PI3K), involved in regulating cell metabolism, proliferation, size, migration, angiogenesis, and survival (Fruman et al., 2017). Mutations in PIK3CA are among the most frequent in solid tumors, and especially in estrogen receptor (ER)-positive breast cancer (Ellis and Perou, 2013; Pereira et al., 2016; Razavi et al., 2018). Small molecule α isoform-specific PI3K inhibitors are currently in clinical trials in breast cancer (Hanker et al., 2019), with alpelisib (PIQRAY, BYL-719), in combination with fulvestrant, having been approved for treatment of hormone receptor (HR)-positive, HER2-negative, *PIK3CA*-mutated tumors (Brandao et al., 2019). However, the development of resistance could limit the utility of these drugs (Hanker et al., 2019) and there is a need to identify biomarkers that could predict efficacy in individual patients.

Inhibition of PI3K signaling can inhibit tumor glycolysis by inhibiting release of aldolase from the cytoskeleton (Hu et al., 2016), suppressing membrane localization of glucose transporter 1 (Makinoshima et al., 2015), and by lowering the levels of c-Myc (Kalkat et al., 2017) and hypoxia-inducible factor 1 (HIF-1α) (Zhong et al., 2000), which drive expression of most of the glycolytic enzymes, including the A isoform of lactate dehydrogenase (LDHA) and hexokinase 2 (HK-II) (Dang et al., 2009). The activities of these enzymes, and therefore inhibition of PI3K, can be assessed using metabolic imaging; for example, PET measurements of the uptake, phosphorylation, and trapping of the glucose analog, 2-deoxy-2-[^18^F]fluorodeoxyglucose ([^18^F]FDG), catalyzed by HK-II, and ^13^C magnetic resonance spectroscopy (MRS) measurements of hyperpolarized ^13^C label exchange between [1-^13^C]pyruvate and the endogenous tumor lactate pool, catalyzed by LDHA (Brindle, 2008). [^18^F]FDG-PET measurements have been used in several early-phase clinical trials of PI3K inhibitors in breast cancer (Hanker et al., 2019), and ^13^C MRS measurements of hyperpolarized [1-^13^C]pyruvate metabolism detected PI3K inhibition in preclinical studies in glioblastoma (Venkatesh et al., 2012) and breast cancer models (Ward et al., 2010). Imaging with hyperpolarized [1-^13^C]pyruvate has translated to the clinic, with initial studies in prostate cancer (Nelson et al., 2013), and more recently in breast cancer (Gallagher et al., 2020).

Here we investigated the potential of these clinical imaging methods for detecting early response and resistance to PI3Kα inhibition in *PIK3CA*-driven ER^+^ breast cancer models.

## Results

### Early Responses to a PI3Kα Inhibitor Can Be Detected Using Metabolic Imaging With Hyperpolarized [1-^13^C]Pyruvate

**Figure 1:**
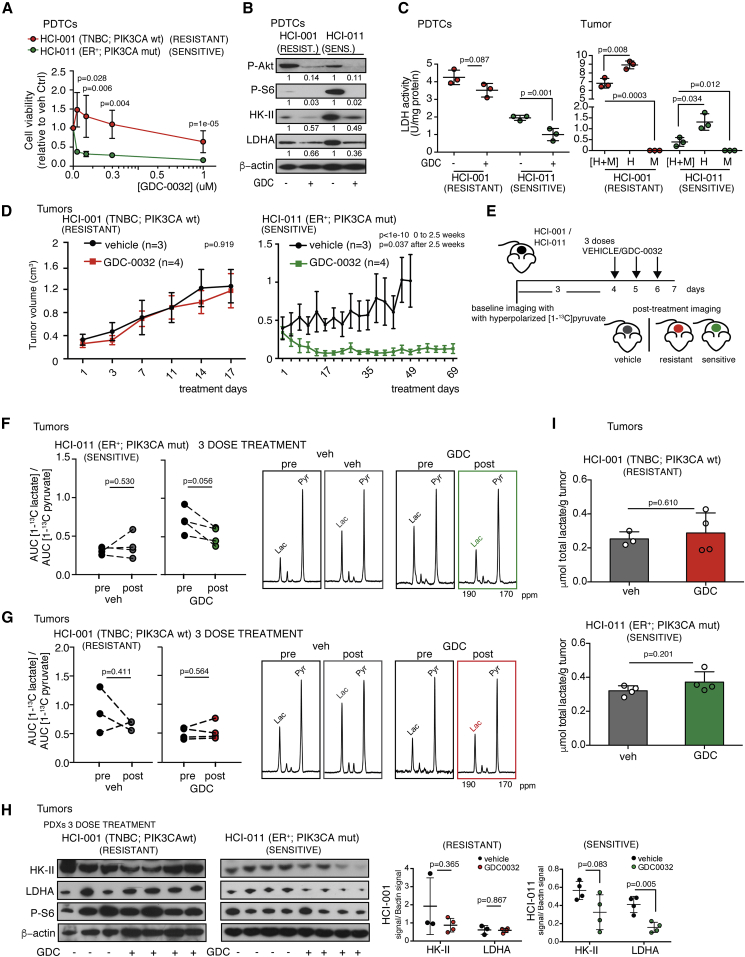
Resistance to a PI3Kα Inhibitor Can Be Detected Using Metabolic Imaging with Hyperpolarized [1-^13^C]pyruvate (A) Cell viability following treatment of HCI-001 and HCI-011 PDTCs with GDC-0032 for 120 h. Mean ± SEM (n = 3 or 5 technical replicates). p values were defined using two-sided Wald t tests. (B) Immunoblot of indicated proteins in lysates of PDTCs treated for 72 h. (C) Left: LDH activity in PDTCs. Right: LDH activity (U/mg protein) in cells isolated from disaggregated tumors. H, human breast cancer epithelial cells; M, mouse stomal cells. Mean ± standard deviation (n = 3 technical replicates). p values were defined using two-sided Welch's t tests. (D) Mean tumor volumes (cm^3^) ± SEM following treatment with vehicle (n = 3) or GDC-0032 (n = 4). p values were defined using two-sided Wald t tests. (E) Protocol for measurements with hyperpolarized [1-^13^C]pyruvate. (F) Changes in [1-^13^C]lactate/[1-^13^C]pyruvate signal ratios in HCI-011 xenografts, following short-term treatment (n = 4 each). Representative ^13^C spectra. (G) Changes in [1-^13^C]lactate/[1-^13^C]pyruvate signal ratios in HCI-001 xenografts following short-term treatment (n = 3 or 4). Representative ^13^C spectra. (H) Immunoblots of HCI-001 and HCI-011 tumors and quantification of HK-II and LDHA following short-term treatment. Mean ± standard deviation (n = 3 or 4). p values were calculated using two-sided Welch's t tests. See also Figure S1. (I) Lactate concentrations (μmol/g tumor) measured by ^1^H NMR. Mean ± standard deviation (n = 3 or 4). p values were calculated using two-sided Welch's t tests.

Resistance to PI3K inhibitors poses a significant clinical challenge in breast cancer and other malignancies. GDC-0032 (taselisib) has greater selectivity for mutant PI3Kα isoforms and is effective in inhibiting proliferation of p110α-mutant breast cancer cell lines *in vitro* and the growth of human breast cancer xenograft models harboring *PIK3CA* mutations *in vivo* (Dickler et al., 2018; Juric et al., 2017; Ndubaku et al., 2013). Consistent with these observations, luminal ER^+^ *PIK3CA*-mutant HCI-011 patient-derived breast cancer cells (PDTCs) were sensitive to short-term treatment with GDC-0032, while triple-negative breast cancer (TNBC) *PIK3CA* wild-type (wt) HCI-001 PDTCs were resistant (Figure 1A). Both showed evidence of drug target engagement with decreased Akt (Ser473) and S6 (Ser 235/236) phosphorylation (Figure 1B). We investigated the effects of GDC-0032 on expression of HK-II in the upper part of the glycolytic pathway, and LDHA in the lower part. HK-II expression was decreased after treatment in both cell lines, whereas the drug-resistant HCI-001 PDTCs showed sustained LDHA protein expression and activity (Figures 1B and 1C).

Next, we implanted HCI-001 and HCI-011 patient-derived breast tumor fragments subcutaneously in NSG female mice. Long-term GDC-0032 treatment had no effect on the growth of HCI-001 patient-derived xenografts (PDXs) but produced a rapid and marked reduction in the size of HCI-011 PDXs (Figure 1D). The rate of hyperpolarized ^13^C label exchange between pyruvate and lactate was assessed by calculating the ratio of the areas under the pyruvate and lactate labeling curves (AUCs) (Hill et al., 2013). After three doses of GDC-0032 (Figure 1E) ^13^C label flux was decreased in the HCI-011 PDXs (Figure 1F) before there were detectable changes in tumor volume (Figure S1A), but not in the drug-resistant HCI-001 PDXs (Figure. 1G). This could be explained by a 60% decrease in LDHA protein in the HCI-011 tumors, which was not observed in the drug-resistant HCI-001 tumors (Figure 1H). Disaggregation of the untreated tumors and flow cytometric sorting of FISH-labeled human epithelial and mouse stromal cells showed that mouse stromal cells constituted less than 10% of the total cell number (Figures S1B and S1C). Measurements of lactate dehydrogenase (LDH) activity showed that this was predominantly in the tumor breast epithelial cells (Figure 1C). Lactate concentration can also influence hyperpolarized ^13^C label flux (Witney et al., 2011); however, there were no significant changes in lactate concentration in drug-sensitive or drug-resistant tumors post treatment with GDC-0032 (Figure 1I). This was consistent with there being no significant changes in HK-II expression, which was unchanged in the drug-resistant HCI-001 tumors following treatment, and was decreased in only two out of four drug-sensitive HCI-011 tumors. Thus GDC-0032 inhibition of LDHA expression in drug-sensitive tumor cells can be detected *in vivo* through decreased ^13^C label exchange between hyperpolarized [1-^13^C]pyruvate and the endogenous lactate pool.

### Imaging With Hyperpolarized [1-^13^C]Pyruvate Can Detect Induced Resistance to PI3Kα Inhibition

Loss of the tumor suppressor *PTEN* led to resistance to the PI3Kα inhibitor BYL-719 (alpelisib) in a breast cancer patient with metastatic breast cancer bearing an activating *PIK3CA*-mutation (Juric et al., 2015). The drug-resistant PDX model, HCI-001, was also *PTEN* null. We therefore knocked down PTEN expression, using a small hairpin RNA (shRNA) mir-based system (Fellmann et al., 2013) (PTEN KD), or knocked-out PTEN expression using CRISPR-Cas9 (PTEN KO), in two ER^+^ *PIK3CA*-mutant breast cancer cell lines, T47D (H1047R) and MCF7 (E545K) (Figures S2A and S2B). Response to GDC-0032 was altered by decreased PTEN expression (Figure S2C). There was higher Akt and S6 phosphorylation (Figure S2B) and induction of drug resistance (Figure S2C) in the PTEN KO and PTEN KD T47D and MCF7 cell lines compared with controls. There was also less inhibition of Akt and S6 phosphorylation in the T47D PTEN KO cells following drug treatment (Figure S2B). High residual tumor S6 phosphorylation has been positively correlated with intrinsic or acquired resistance to PI3Kα inhibitors (Elkabets et al., 2013). GDC-0032 decreased LDH activity in both parental cell lines, but this was blunted in cells with decreased or no PTEN expression (Figure S2D). GDC-0032 treatment had little effect on [^18^F]FDG uptake, regardless of PTEN status (Figure S2E).

**Figure 2:**
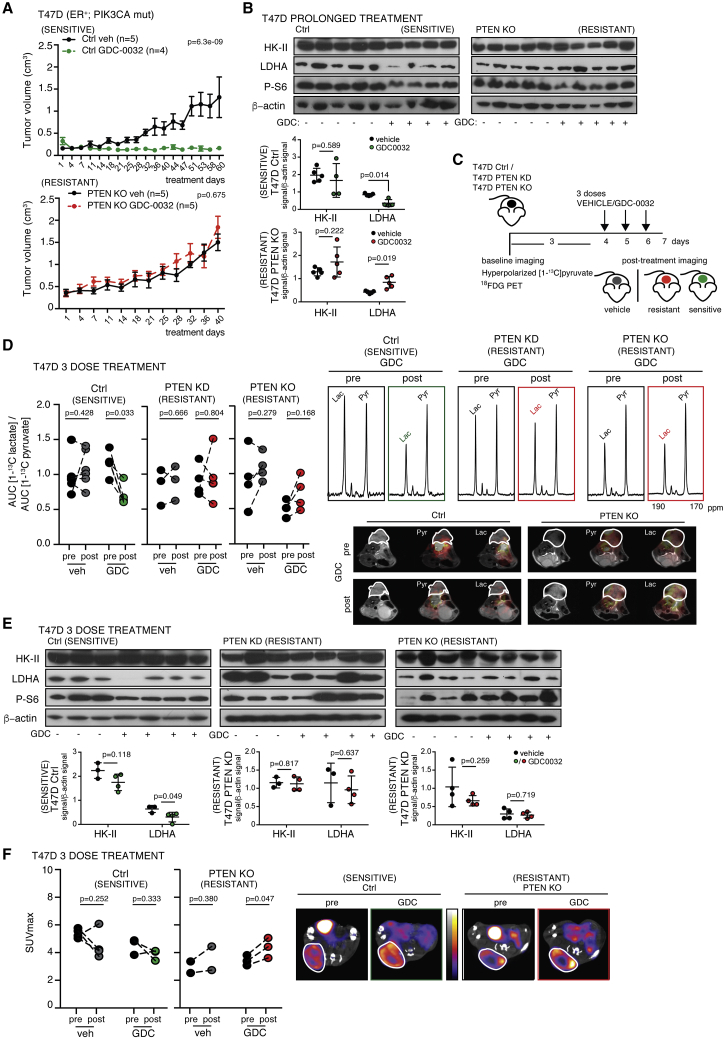
Imaging with Hyperpolarized [1-^13^C]pyruvate Detects Sensitivity and Induced Resistance to PI3Kα Inhibition (A) Mean tumor volume (cm^3^) ± SEM for PTEN wt (Ctrl) and PTEN KO xenografts, following prolonged treatment. p values were defined using two-sided Wald t tests. (B) Immunoblot of tumor lysates from (A). Mean ± standard deviation (n = 4 or 5). p values were defined using two-sided Welch's t tests. (C) Imaging protocol. (D) [1-^13^C]lactate/[1-^13^C]pyruvate AUC ratios (drug vehicle: Ctrl, n = 7; PTEN KD, n = 3; PTEN KO, n = 4. GDC-0032: Ctrl, n = 5; PTEN KD, n = 4; PTEN KO, n = 4). Representative ^13^C spectra and false-color images of the [1-^13^C]lactate and [1-^13^C]pyruvate signals superimposed on T_2_-weighted ^1^H images, where the lactate signal is scaled to ten times that of the pyruvate signal. p values were defined using two-sided Student's paired t tests. (E) Immunoblots of lysates from (D). Mean ± standard deviation (n = 3 or 4). p values were defined using two-sided Welch's t tests. (F) PET measurements of [^18^F]FDG uptake (maximal standardized uptake value [SUV_max_]) in the T47D PTEN wt (Ctrl, n = 3 or 4) and PTEN KO tumors (n = 2 or 3), short-term treatment. Representative [^18^F]FDG-PET/computed tomography (CT) images before and after treatment. The CT images are shown in Hounsfield units (−1000 to 3500) and the PET images in MBq/mL (0.1–5). p values were defined using two-sided Student's paired t tests. See also Figure S2.

Next, we implanted the PTEN KO, PTEN KD and PTEN wt (control [Ctrl]) T47D cell lines into female NSG mice. PTEN knockdown in the tumors was confirmed by measurements of PTEN mRNA (Figure S2F). Xenografted tumors not expressing PTEN grew more rapidly and were insensitive to prolonged treatment with GDC-0032 (Figure 2A). The drug-sensitive T47D PTEN wt tumors expressed less LDHA protein following prolonged treatment with GDC-0032, which was unchanged in the resistant PTEN KO tumors (Figure 2B). There were no significant changes in HK-II protein regardless of PTEN status (Figure 2B). The decrease in LDHA activity in the drug-sensitive PTEN wt tumors could be detected non-invasively *in vivo* after only three doses of GDC-0032 (Figure 2C) as a decrease in lactate labeling following injection of hyperpolarized [1-^13^C]pyruvate (Figure 2D). This treatment protocol had no effect on lactate labeling in drug-resistant T47D PTEN KO and PTEN KD tumors (Figure 2D). The decrease in lactate labeling was observed before there was a change in tumor growth (Figure S2G), which, for the drug-sensitive PTEN wt tumors, became apparent after 18 days of treatment (Figure 2A). LDHA protein concentration was reduced in the GDC-0032-sensitive tumors (T47D Ctrl), whereas there was sustained expression in the drug-resistant tumors (T47D PTEN KO and PTEN KD) (Figure 2E). Consistent with the absence of an effect on HK-II (Figure 2E), PET measurements failed to show any change in [^18^F]FDG uptake post drug treatment, regardless of PTEN status (Figure 2F). There was decreased Akt phosphorylation in the T47D Ctrl tumors and less so in the T47D PTEN KO tumors following treatment (Figure S2H).

### Combination Treatments That Overcome Resistance to PI3Kα-Specific Inhibition Resulted in Decreased LDHA Protein Expression and Enzyme Activity

**Figure 3:**
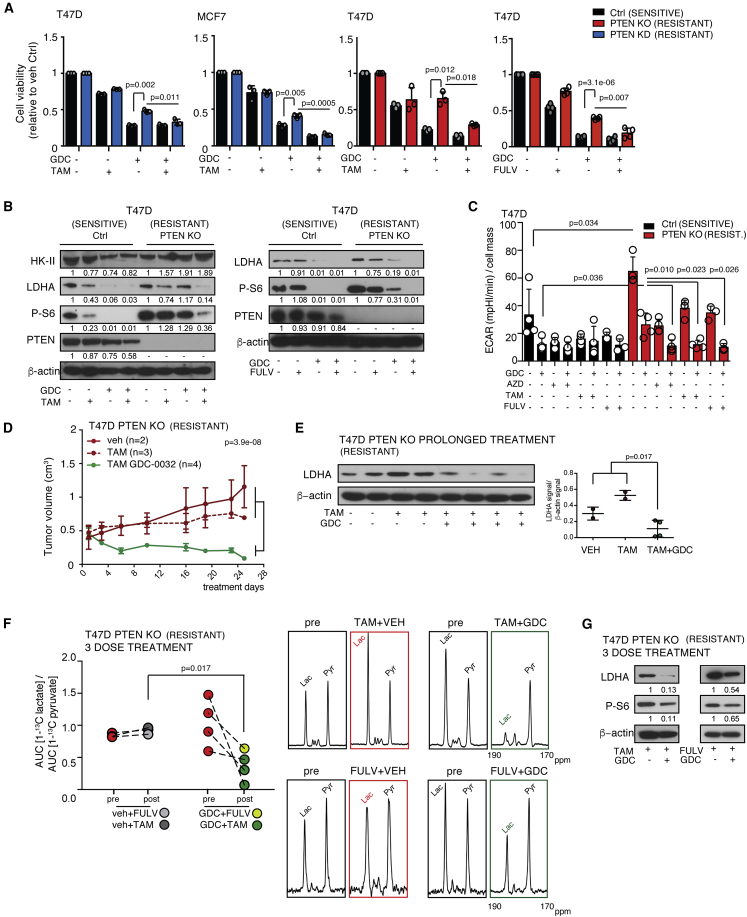
Combination Treatments that Overcome Resistance to PI3Kα-Inhibition Result in Decreased LDHA Expression (A) Viability of PTEN wt (Ctrl) and MCF7 PTEN KD, T47D KO, and T47D PTEN KO cells treated for 120 h. Mean ± standard deviation (n = 3 to 4, 5 technical replicates). p values were calculated using two-sided Welch's t tests on the averages of technical replicates. (B) Immunoblots of lysates of cells treated for 72 h. (C) Total extracellular acidification rate (basal extracellular acidification rate [ECAR], in (mpH/min)/cell mass) measured using a Seahorse instrument after pre-incubation with drugs in the indicated combinations for 72 h. ECAR is presented as mean ± standard deviation of experimental quintuplicates. p values were calculated using two-sided Welch's t tests on the averages of technical replicates. (D) Mean tumor volume (cm^3^) ± SEM of PTEN KO T47D xenografts. Vehicle, n = 2; vehicle + tamoxifen, n = 3; GDC-0032 and tamoxifen, n = 4. p values were defined using two-sided Wald t tests. (E) Immunoblot of tumor lysates from (D). One tumor sample treated with tamoxifen for only 10 days was excluded from the analysis. Mean ± standard deviation (n = 2 to 4). p values were defined using two-sided Welch's t tests. (F) [1-^13^C]lactate/[1-^13^C]pyruvate AUC ratios in T47D PTEN KO xenografts, short-term treatment. Vehicle plus hormone therapy, n = 3; hormone therapy + GDC-0032, n = 4. p values were calculated using two-sided Welch's t tests. Representative ^13^C spectra. (G) Immunoblots of lysates from (F). See also Figure S3.

Next, we explored the effects of PI3Kα inhibition in combination with endocrine therapy. Inhibition of PI3K signaling in HR-positive breast cancer results in upregulation of ER-dependent function (Bosch et al., 2015), providing a basis for dual PI3K and ER inhibition. Combination of GDC-0032 with tamoxifen or fulvestrant decreased cell viability, compared with either agent alone, in cells rendered GDC-0032 resistant by PTEN deletion (T47D PTEN KO) or PTEN knockdown (T47D and MCF7 PTEN KD) (Figure 3A). In the absence of PTEN, cells can become dependent on the p110β isoform of PI3K (PI3Kβ) (Edgar et al., 2010). Combining the PI3Kβ inhibitor, AZD-6482, with GDC-0032 or BYL-719 also reverted resistance in single-agent-resistant cells (Figure S3A). GDC-0032, and its combination with tamoxifen, had little effect on HK-II in T47D Ctrl and PTEN KO cells (Figure 3B), consistent with only small changes in [^18^F]FDG uptake (Figure S3B), whereas LDHA protein concentration was decreased in drug-sensitive cells (T47D Ctrl) by GDC-0032 treatment and by its combination with tamoxifen or fulvestrant in drug-resistant cells (Figure 3B). LDHA activity was decreased by treatment with PI3Kα inhibitors or PI3Kβ inhibitors in drug-sensitive cells (T47D Ctrl) and by combination of the PI3Kα inhibitors with the PI3Kβ inhibitor in drug-resistant cells (T47D PTEN KO) (Figure S3C).

Although there was no effect of GDC-0032 on lactate concentration in the PDXs (Figure 1I) nor of GDC-0032, or combinations of GDC-0032 with other drugs, on HK-II, [^18^F]FDG uptake, the monocarboxylate (MCT1 and MCT4) and glucose (GLUT1 and GLUT3) transporters (Figure S3D) in T47D Ctrl and PTEN KO cells, there was nevertheless an effect of these drugs on glycolytic flux in the cells in culture. The total extracellular acidification rate was decreased in T47D Ctrl cells by tamoxifen, fulvestrant, GDC-0032, and the PI3Kβ inhibitor AZD-6482, and there was some further inhibition with combinations of these drugs (Figure 3C). However, this inhibition was decreased in the drug-resistant T47D PTEN KO cells, with only the drug combinations GDC-0032+AZD-6482, GDC-0032 + tamoxifen and GDC-0032 + fulvestrant giving a decrease comparable with that observed in the drug-sensitive controls.

Next we examined the effect of drug combinations in implanted tumors. T47D PTEN KO tumors were insensitive to tamoxifen treatment but combination with GDC-0032 inhibited their growth (Figure 3D). Loss of PTEN expression in ER^+^ breast cancer has been associated with relapse following tamoxifen treatment (Shoman et al., 2005). Following prolonged treatment with tamoxifen and GDC-0032, T47D PTEN KO tumors expressed less LDHA, which was unchanged in tumors treated with tamoxifen alone (Figure 3E). Tamoxifen + GDC-0032 (3 doses each) or fulvestrant + GDC-0032 (one dose of fulvestrant plus three doses of GDC-0032) also produced marked decreases in lactate labeling, following injection of hyperpolarized [1-^13^C]pyruvate, compared with endocrine therapy alone (Figure 3F). This short-term treatment also decreased S6 phosphorylation and the LDHA concentration (Figure 3G).

We determined whether LDHA played a role in response and resistance to PI3Kα inhibitors by performing a loss-of-function assay. However, inducible short-term LDHA knockdown, or inhibition with FX-11 (Edgar et al., 2010), did not enhance drug sensitivity in GDC-0032-resistant T47D PTEN KO cells (Figures S3E–S3G).

### Effective PI3Kα Inhibition Lowered Tumor FOXM1, Whereas Acquisition of Resistance Resulted in Persistent FOXM1 and LDHA Expression

**Figure 4:**
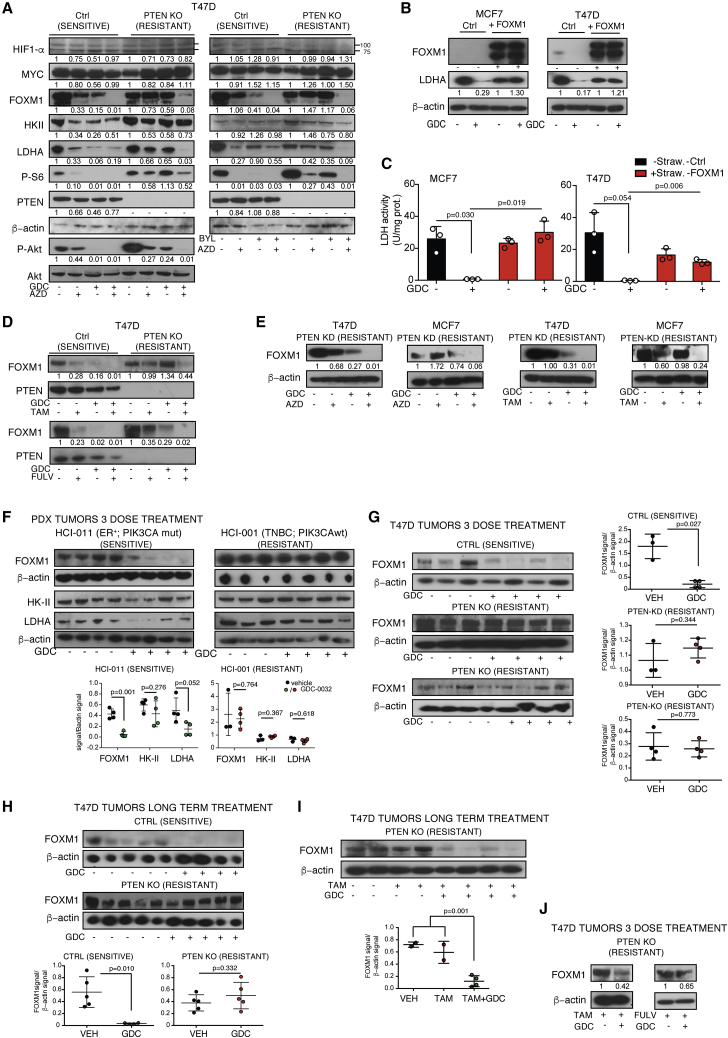
PI3Kα Inhibition Decreases FOXM1 Expression, Whereas Drug-Resistant Tumors Show Sustained Expression (A) Immunoblots of lysates from PTEN wt (Ctrl) and PTEN KO T47D cells, treated for 72 h. (B) Immunoblots of lysates from MCF7 and T47D cells and cells co-expressing FOXM1C and mStrawberry following treatment for 96 h. The upper FOXM1C band is a FOXM1-mStrawberry fusion protein. (C) LDH activity in cells from (B) following treatment for 96 h. Mean ± standard deviation (n = 3). p values were calculated using two-sided Welch's t tests. (D) Immunoblots of lysates of the indicated cells treated for 72 h. (E) Immunoblots of lysates from the indicated cells following treatment for 72 h. (F) Immunoblots of lysates from the indicated tumors, short-term treatment. Mean ± standard deviation (n = 3 or 4). p values were calculated using two-sided Welch's t tests. (G) Immunoblots of lysates from the indicated tumors, short-term treatment. Mean ± standard deviation (n = 3 or 4). p values were calculated using two-sided Welch's t tests. (H) Immunoblots of lysates from the indicated tumors, long-term treatment. Mean ± standard deviation (n = 4 or 5). p values were calculated using two-sided Welch's t tests. (I) Immunoblot of lysates from the indicated tumors, long-term treatment. Mean ± standard deviation (n = 2 to 4). p values were calculated using two-sided Welch's t tests. (J) Immunoblots of lysates from the indicated tumors, short-term treatment. See also Figure S4.

The promoter regions of HK-II and LDHA contain consensus binding sites for HIF-1α and c-Myc (Dang et al., 2009; Valvona et al., 2016). However, their expression was unchanged after single-agent treatment in T47D PTEN wt cells (Ctrl) or combined PI3Kα and β blockade in PTEN KO cells (Figure 4A). This explains why HK-II was unchanged but not why there was a decrease in LDHA expression. LDHA is also transcriptionally regulated by FOXM1 (Cui et al., 2014), one of the most commonly upregulated genes in human solid tumors (Koo et al., 2012). We confirmed binding of FOXM1 to the LDHA promoter in T47D Ctrl and PTEN KO cells, and that this was decreased after GDC-0032 treatment in the Ctrl cells (Figure S4A). FOXM1C overexpression in MCF7 or T47D cells resulted in sustained LDHA protein concentration and enzyme activity in GDC-0032-treated cells (Figures 4B and 4C). FOXM1 mRNA and protein were decreased following GDC-0032, BYL-719, AZD-6482, tamoxifen, or fulvestrant treatment of T47D and MCF7 Ctrl cells (Figures 4A, 4D, and S4B) and after combinatorial treatments (PI3Kα inhibitor [GDC-0032, BYL-719] with a β inhibitor [AZD-6482]) or endocrine therapy (tamoxifen or fulvestrant) of single-agent-resistant T47D PTEN KO or PTEN KD cells (Figures 4A, 4D, 4E, and S4B). In MCF7 and T47D cells rendered drug resistant by prolonged culture with PI3Kα inhibitors (Figure S4C), the concentration of FOXM1 protein (Figure S4D) and LDHA protein and activity (Figures S4E and S4F) were maintained following treatment with GDC-0032 or BYL-719. Treatment of drug-resistant TNBC *PIK3CA* wt MDAMB-231 and MDAMB-468 cells with GDC-0032 had no effect on cell viability (Figure S4G) or the concentrations of FOXM1 and LDHA (Figure S4H).

**Figure 5:**
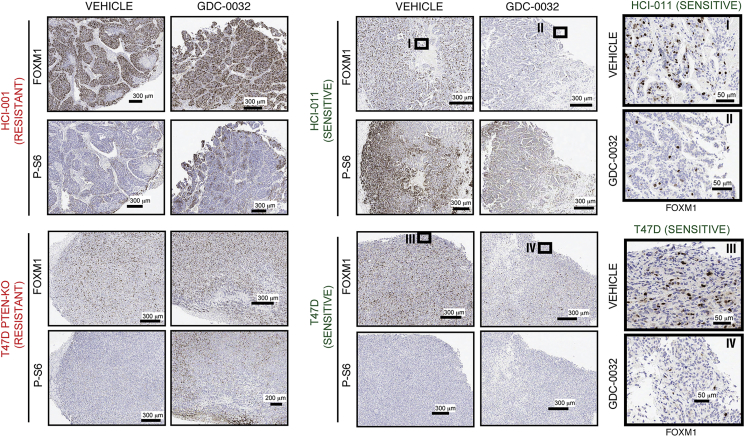
Immunohistochemical Analysis of Persistent FOXM1 Expression in Drug-Resistant Tumors Following Treatment with GDC-0032 Representative formalin-fixed paraffin-embedded (FFPE) sections obtained from tumors after short-term treatment stained for FOXM1, sections obtained from tumors after short-term treatment. Images were captured at ×40 magnification. Boxes labeled I–IV are from the indicated areas.

Next, we determined the effect of PI3Kα inhibition on FOXM1 in implanted tumors. FOXM1 was decreased in the GDC-0032-sensitive HCI-011 PDX, but not in the drug-resistant HCI-001 PDX, after short-term GDC-0032 treatment (Figure 4F). FOXM1 was also lowered in the T47D drug-sensitive tumors, but not in the PTEN KO or PTEN KD drug-resistant tumors, following short-term (Figure 4G) or prolonged GDC-0032 treatment (Figure 4H) and was lowered in the T47D PTEN KO tumors following long-term treatment with GDC-0032 plus tamoxifen (Figure 4I), or short-term treatment with GDC-0032 plus tamoxifen or GDC-0032 plus fulvestrant (Figure 4J). Immunohistochemical staining (Figure S4I) confirmed sustained FOXM1 expression after treatment of resistant tumors (HCI001 and T47D PTEN KO) with GDC-0032 and decreased FOXM1 in sensitive tumors (HCI011 and T47D) (Figure 5).

**Figure 6:**
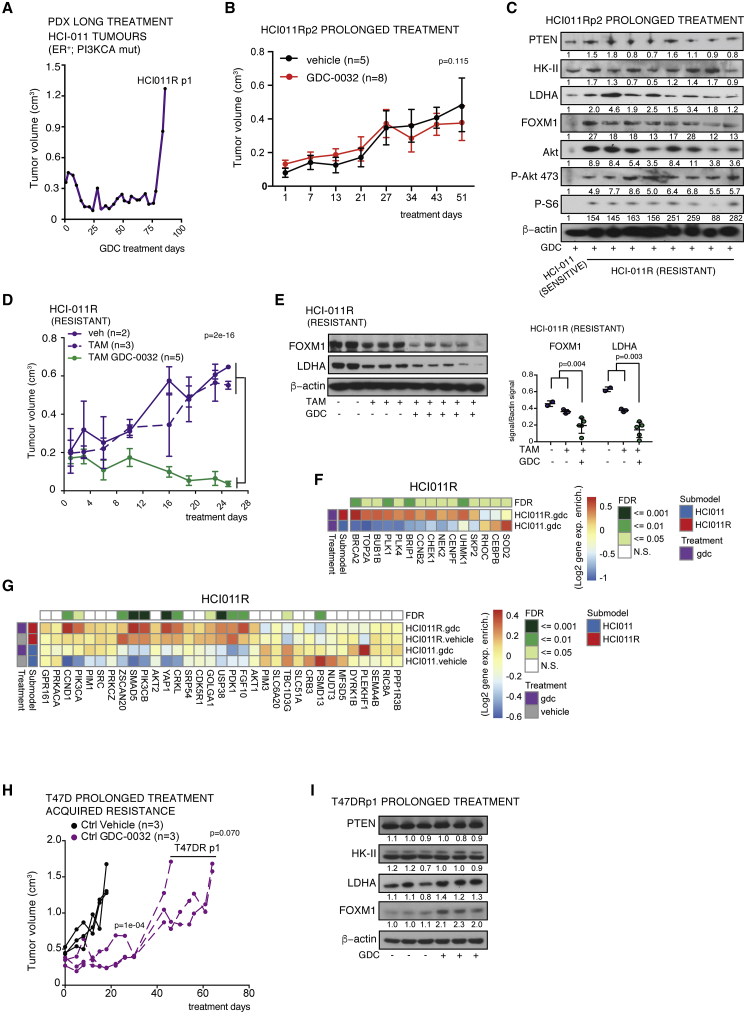
Persistent FOXM1 Expression in Breast Cancer PDXs That Have Acquired Resistance to PI3Kα Inhibition (A) Relapse of an HCI-011 PDX after treatment with GDC-0032 (HCI-011R). (B) Mean tumor volumes (cm^3^) ± SEM of HCI011R xenografts receiving drug vehicle (n = 5) or GDC-0032 (n = 8). p values were defined using two-sided Wald t tests. (C) Immunoblots of lysates from (B) and from an HCI-011 tumor. (D) Mean tumor volumes (cm^3^) ± SEM of HCI011R xenografts treated with drug vehicle (n = 2), drug vehicle plus tamoxifen (n = 3), and tamoxifen plus GDC-0032 (n = 5). p values were defined using two-sided Wald t tests. (E) Immunoblots of lysates from (D). Mean ± standard deviation (n = 2 to 4). p values were calculated using two-sided Welch's t tests. (F) Gene expression (Log2) enrichment (heatmap: red, positive; white, neutral; blue, negative) of FOXM1 gene targets in HCI-011 (n = 4) and in HCI-011R xenografts (n = 8). The top row shows false discovery rate (FDR)-adjusted p values. (G) Gene expression (Log2) enrichment of genes mediating resistance to BYL-719 in HCI-011 (n = 4 for vehicle and drug) and in HCI-011R (n = 5 vehicle, n = 8 GDC-0032). FDR-adjusted p values of drug-resistant versus drug-sensitive differential expression are shown in the top row. (H) Mean tumor volume (cm^3^) ± SEM of drug-resistant T47DR tumors after long-term treatment with GDC-0032. p values were defined using two-sided Wald t tests. (I) Immunoblot of tumors lysates from (H). See also Figure S5.

Following prolonged GDC-0032 treatment of HCI-011 PDXs (Figure 1D) one (HCI-011R) out of the four treated tumors relapsed after 86 days (Figure 6A). We passaged HCI-011R tumor fragments into 13 mice and treated them for an additional 57 days (GDC-0032 or vehicle treated). Although they had not lost PTEN, they remained insensitive to drug treatment (Figure 6B) and showed sustained PI3K signaling following GDC-0032 treatment, as indicated by higher concentrations of phosphorylated S6 and Akt, and sustained expression of FOXM1 and LDHA (Figure 6C). Tumor growth was inhibited by treatment with tamoxifen plus GDC-0032, but not by tamoxifen alone (Figure 6D), and this combined treatment resulted in decreased FOXM1 and LDHA expression (Figure 6E). RNA sequencing (RNA-seq) analysis of the drug-resistant (HCI-011R) compared with the drug-sensitive parent tumor (HCI-011) showed no change in PTEN expression and upregulation of FOXM1 targets, including genes in the G2M checkpoint and E2F target pathways (Figures 6F, S5A, and S5S5B), and upregulation of several genes known to mediate resistance to BYL-719 (Le et al., 2016) and genes that have been listed as driving resistance to PI3K inhibition (Figure S5C) (Hanker et al., 2019), some of which have been shown to affect FOXM1 expression, including *CDK4*, *CDK6*, *FGFR1*, *KRAS*, *PDK1*, *PIK3CB*, and *RICTOR*. We also found upregulation of mTORC1 signaling, consistent with the observed increased concentrations of P-S6 in the drug-resistant tumors (HCI011R and T47D PTENKO) after single-drug treatment. There was upregulation of *PIK3CA* and *PIK3CB* gene expression and of genes that are established as mediating resistance to PI3K inhibitors (Costa et al., 2015; Huw et al., 2013), including *PDK1* (Figure 6G). PDK1 limits sensitivity to PI3Kα inhibition by maintaining mTORC1 activity upon PI3Kα inhibition (Castel et al., 2016), which is consistent with the increased phosphorylated S6 and Akt, as well as total Akt (Figure 6C). There was increased expression of other genes in the HCI-011R PDXs that exert known roles in signaling, such as growth factors (*FGF10*), serine-threonine kinases (*CCND1*), adapter proteins (*CRKL*), transcription factors/cofactors (*SMAD5*, *ZSCAN20*, and *YAP1*), and others (*GOLGA1*, *USP38*) (Figure 6G). These were previously identified as genes mediating resistance to the PI3Kα inhibitor BYL-719 (Le et al., 2016). Prolonged GDC-0032 treatment of drug-sensitive T47D tumors (Figure 2A) also resulted in the development of resistance (Figure 6H), which again could not be explained by loss of PTEN expression, but, as with the other drug-resistant tumors, FOXM1 and LDHA expression were no longer decreased by GDC-0032 treatment (Figure 6I).

### FOXM1 as a Biomarker of Resistance to PI3Kα Inhibitors Can Be Explained by PI3K Signaling-Dependent Phosphorylation of FOXO3a

**Figure 7:**
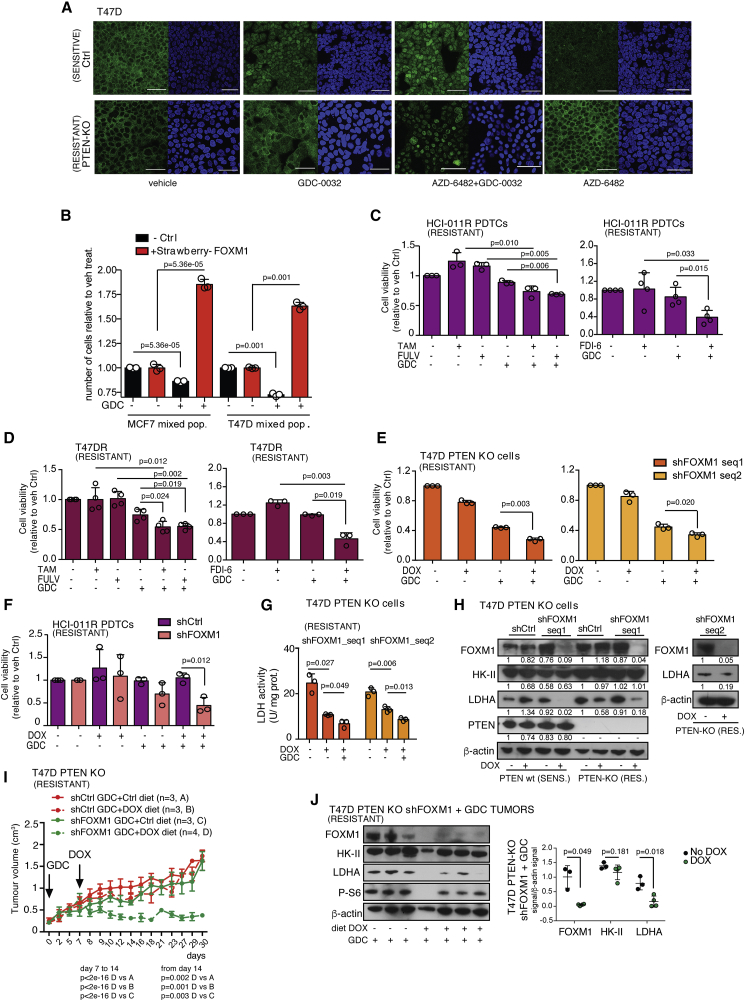
Persistent FOXM1 Expression Contributes to Drug Resistance and Can Be Explained by Cytoplasmic Localization of FOXO3a (A) Representative confocal microscopy images of T47D PTEN wt (Ctrl) and PTEN KO cells drug-treated for 72 h. FOXO-3a staining (green), DAPI staining (blue). (B) Relative number of red fluorescent MCF7 and T47D cells (mStrawberry-positive cells, co-expressing FOXM1C) in mixtures with parental controls following treatment for 120 h. p values were calculated using two-sided Welch's t tests. (C) Viability of HCI-011R PDTCs treated with the indicated drugs for 120 h. Left: viability of HCI-011R PDTCs treated for 120 h. Mean ± standard deviation (five technical replicates, n = 3 experiments). Right: mean ± standard deviation (five technical replicates, n = 4 experiments). p values were calculated using two-sided Welch's t tests on the averages of replicates. (D) Viability of cells dissociated from a T47DR tumor treated with the indicated drugs for 120 h. Left: mean ± standard deviation (five technical replicates, n = 4 experiments). Right: mean ± standard deviation (five technical replicates, n = 3 experiments). p values were calculated using two-sided Welch's t tests on the averages of replicates. (E) Viability of T47D PTEN KO cells expressing FOXM1 targeting sequences (shFOXM1 [sequence 1 or 2]) or a non-targeting control sequence (shCtrl) treated for 120 h. Mean ± standard deviation (n = 5, five technical replicates). p values were calculated using two-sided Welch's t tests on the averages of replicates. (F) Viability of HCI-011R PDTCs expressing doxycycline-inducible shRNA sequences targeting FOXM1 (sequence 1) or a control shRNA and treated for 120 h. Mean ± standard deviation (three technical replicates). p values were calculated using two-sided Welch's t tests on the averages of the replicates. (G) LDH activity in the cells used in (E) and treated for 96 h. Figure shows the mean ± standard deviation (n = 3 experiments). p values were calculated using two-sided Welch's t tests on the averages of replicates. (H) Immunoblots of lysates from cells used in (G). (I) Mean tumor volume (cm^3^) ± SEM of T47D PTEN KO xenografts expressing doxycycline-inducible shFOXM1 (sequence 1) or shCtrl, following prolonged GDC-0032 treatment. One cohort received standard food (shCtrl n = 3 and shFOXM1 n = 3) and the other cohort food plus doxycycline (shCtrl n = 3 and shFOXM1 n = 4; 0.2 g/kg food pellet Harlan D.98186). p values were defined using two-sided Wald t tests. (J) Immunoblot of lysates from tumors in (I). p values were calculated using two-sided Welch's t tests. See also Figure S6.

Transcription of FOXM1 is antagonized by FOXO3a (Nestal de Moraes et al., 2016). Phosphorylation of FOXO3a by Akt, downstream of PI3K signaling, results in its cytoplasmic accumulation and inhibition of its activity (Tzivion et al., 2011). Complete PI3K/Akt/mTOR inhibition by single PI3Kα blockade resulted in FOXO3a nuclear relocation in T47D GDC-0032- and AZD-6482-sensitive cells (Figure 7A), whereas it was mainly cytoplasmic in drug-resistant cells (PTEN KO). Combined PI3Kα and β blockade of the drug-resistant cells resulted in FOXO3a relocation to the nucleus (Figure 7A), and inhibition of FOXM1 expression (Figure 4A). Analysis of the METABRIC (Molecular Taxonomy of Breast Cancer International Consortium) dataset (Figure S6A, data taken from Curtis et al., 2012; Pereira et al., 2016) showed that FOXM1 expression is inversely correlated with PTEN expression, consistent with this model for the PI3K-Akt pathway regulation of FOXM1 activity.

### FOXM1 Overexpression Confers Drug Resistance

We generated MCF7 and T47D cell lines that co-expressed FOXM1C and mStrawberry. After mixing with the parental lines, the relative number of mStrawberry-positive fluorescent cells, which were overexpressing FOXM1C, were increased following GDC-0032 treatment, demonstrating that FOXM1C overexpression facilitates survival under the selection pressure of PI3Kα inhibition (Figure 7B). Viability of drug-resistant HCI-011R and T47DR cells was decreased when GDC-0032 treatment was combined with FDI-6 inhibition of FOXM1 expression (Gormally et al., 2014) and was lower than or comparable with that observed following co-treatment with GDC-0032 and tamoxifen or fulvestrant (Figures 7C and 7D). FOXM1 knockdown in combination with GDC-0032 treatment also reduced cell viability in PI3Kα single-agent-resistant T47D PTEN KO and HCI-011R cells (Figures 7E, 7F, S6B, and S6C), which was accompanied by decreased LDHA mRNA and protein expression and activity, with no change in HK-II expression (Figures 7G, 7H, and S6B). Doxycycline-induced knockdown of FOXM1 expression in combination with GDC-0032 treatment suppressed the growth of T47D PTEN KO GDC-0032-resistant tumors (Figure 7I), which showed decreased FOXM1 and LDHA compared with tumors treated with GCD-0032 alone (Figures 7J and S6D). These results show that FOXM1 knockdown enhances sensitivity to GDC-0032 in drug-resistant cells both *in vitro* and *in vivo*. Analysis of the METABRIC dataset (Curtis et al., 2012) showed that increased FOXM1 expression correlates with poorer survival in *PIK3CA* mutant breast cancer patients (Figure S6E), and that breast cancer-specific survival rate is decreased in ER^+^ breast cancer patients with FOXM1 copy number gain (Figure S6F). The risk-of-recurrence calculated from follow-up data from METABRIC (Rueda et al., 2019), based on *FOXM1* gene expression in the primary tumor, showed that the risk of relapse is increased in ER^+^ breast cancer compared with ER^−^ breast cancer (Figure S6G).

### Clinical Studies

**Figure 8:**
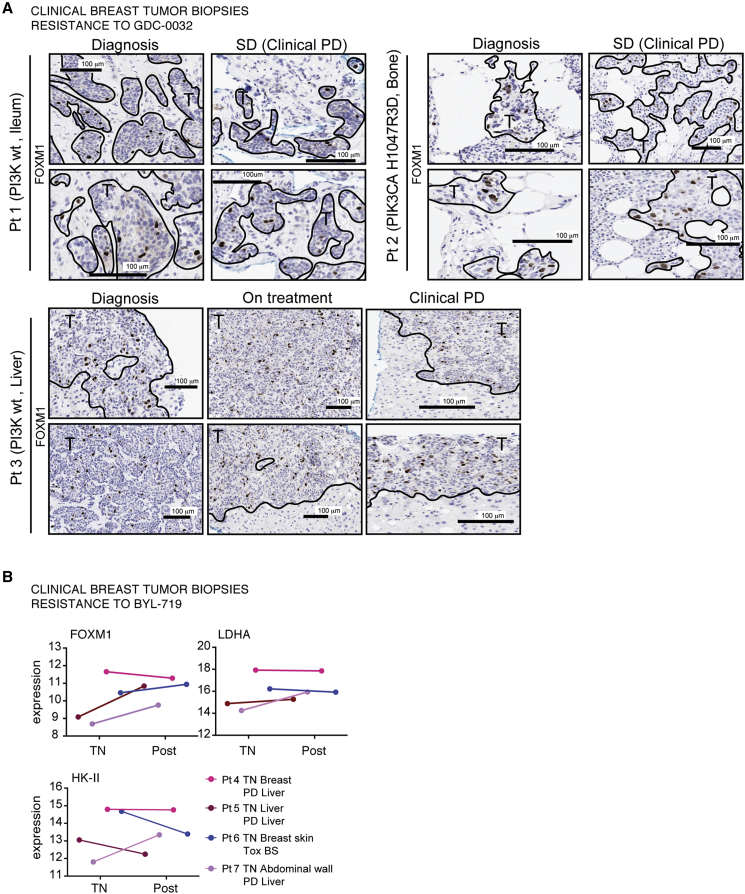
Tumor FOXM1 Expression and Response to PI3Kα Inhibitors in Breast Cancer Patients (A) FFPE needle biopsies from three patients with advanced breast cancer enrolled in the POSEIDON trial (Baird et al., 2019) were stained for FOXM1. All three patients progressed on treatment with GDC-0032 and tamoxifen (progressive disease [PD]). Patient 1: metastatic tumor (no *PIK3CA* mutation) and normal ileum taken on diagnosis and when assessed to have stable disease (SD). Patient 2: metastatic tumor (*PIK3CA* mutation, exon 20) and normal bone taken on diagnosis and SD. Patient 3: metastatic tumor (no *PIK3CA* mutation) and normal liver taken on diagnosis, during treatment, and when diagnosed with PD. Images were captured at ×20 magnification. (B) Relative expression of genes in a collection of paired biopsies from treatment naive (TN) and post-relapse (Post) breast cancer patients treated with BYL-719 (re-analysis of data shown in Le et al., 2016). Patient 4: E545K PIK3CA mutant invasive lobular carcinoma, biopsied in breast when TN and, following disease progression, a metastasis in the liver. Patient 5: E545K PIK3CA mutant invasive ductal carcinoma (IDC), biopsied when TN and post treatment from a metastasis in the liver. Patient 6: H1047R PIK3CA mutant IDC, biopsied when TN and post treatment from a tumor in the breast skin (BS). Patient 7: H1047R PIK3CA mutant IDC, biopsied when TN from a tumor in the abdominal wall and from a liver tumor post treatment.

We investigated whether there was a correlation between inhibition of FOXM1 expression and response to GDC-0032 in patients with ER^+^ metastatic breast cancer. FOXM1 expression was determined by immunohistochemical analysis of biopsies obtained from three patients with advanced breast cancer enrolled in the POSEIDON phase Ib trial, which was designed to assess the efficacy of treatment with GDC-0032 plus tamoxifen (Baird et al., 2019) (Figure 8A). All three patients were eventually diagnosed as having progressive disease since the treatment regime failed. In all of these patients we detected similar FOXM1 expression in serial biopsies (Figure 8A). We also reanalyzed RNA expression in paired biopsies, treatment naive and post relapse, from breast cancer patients treated with BYL-719 (Le et al., 2016) (Figure 8B). Samples from responding patients were unavailable to us as these patients were not biopsied or had inadequate tumor tissue for RNA sequencing. *FOXM1* gene expression was upregulated in three and unchanged in one of the post-relapse biopsies, LDHA gene expression was maintained in three and slightly upregulated in one, whereas HK-II was more variable, with two patients showing downregulation, one was upregulated, and one showed no change in mRNA expression.

## Discussion

We have shown that inhibition of PI3K signaling with a PI3Kα inhibitor (GDC-0032) decreased hyperpolarized ^13^C label exchange between injected [1-^13^C]pyruvate and the endogenous tumor lactate pool. This effect was detected after three drug doses before there was an apparent change in tumor growth rate. Decreased lactate labeling could be explained by decreased FOXM1 expression, resulting in decreased LDHA expression. Decreased lactate labeling could also have resulted from a decrease in lactate concentration (Kettunen et al., 2010; Witney et al., 2011). However, there were no detectable changes in lactate concentration following GDC-0032 treatment of drug-sensitive tumors. There was also little change in c-Myc or HIF-1α, which explains the absence of change in HK-II expression and the much smaller changes in [^18^F]FDG uptake. Although a phase I dose-escalation study of GDC-0032 showed metabolic responses with [^18^F]FDG/PET, these measurements were made when there already appeared to be a reduction in tumor size (Juric et al., 2017). The best time to detect response using [^18^F]FDG/PET is currently unclear, and drug-induced inhibition of [^18^F]FDG uptake has been only partial in several early-phase clinical studies of PI3K inhibitors (Hanker et al., 2019). Treatment of a murine breast cancer model with a PI3Kα inhibitor (BYL-719) resulted in only a small reduction in [^18^F]FDG uptake (Hu et al., 2016) but a 40% to 50% decrease in the conversion of hyperpolarized [1-^13^C]pyruvate to lactate, which in this study was attributed to inhibition of the glycolytic enzyme aldolase and a resulting decrease in NADH concentration. Our results have confirmed that [^18^F]FDG uptake is relatively insensitive to early PI3Kα inhibition, but that the significant decrease in hyperpolarized ^13^C label exchange between pyruvate and lactate can be explained by decreased LDHA expression resulting from decreased FOXM1 expression. Treatment of a TNBC model, MDA-MB231, with a pan PI3K inhibitor (LY294002) (Ward et al., 2010) also decreased hyperpolarized ^13^C label exchange between pyruvate and lactate. However, in this case this was explained by decreased LDHA expression resulting from a reduction in HIF-1α expression, which should also decrease HK-II expression (Denko, 2008) and lower [^18^F]FDG uptake. The pan PI3K inhibitors, LY294002 and wortmannin, have been shown to inhibit HIF expression (Zhong et al., 2000).

FOXM1 expression is negatively regulated by Forkhead box O3 (FOXO3a), which is phosphorylated by the PI3K-Akt pathway (Yao et al., 2017). Phosphorylation results in its cytoplasmic accumulation, inhibition of its activity, and increased FOXM1 expression (Tzivion et al., 2011). PI3K, Akt, and dual PI3K/mTOR inhibitors have been shown to enhance FOXO3a nuclear localization in breast cancer cells (Serra et al., 2011) and we have shown here that effective PI3Kα inhibition resulted in FOXO3a nuclear relocation. This mechanism for the regulation of FOXM1 expression means that any drug resistance mechanism that results in sustained PI3K signaling will lead to persistent FOXM1 expression following drug treatment and, since in ER^+^ breast cancer cells FOXM1 drives expression of LDHA, will lead to sustained exchange of hyperpolarized ^13^C label between pyruvate and lactate.

The expression of FOXM1 was markedly decreased in the ER^+^ *PIK3CA*-mutant PI3Kα inhibitor-sensitive breast tumor models (HCI-011, MCF7, T47D) following PI3Kα inhibition, whereas its expression was unchanged in the *PIK3CA*-wt tumor models (MDAMB231, MDAMB468, HCI-001) that were resistant to PI3Kα inhibitors. We also engineered six models of acquired resistance, three by modulation of PTEN expression (MCF7-PTEN KD, T47D-PTEN KD, T47D-PTEN KO) (Juric et al., 2015), and three by prolonged drug treatment (MCF7R, T47DR, HCI-011R). RNA-seq analysis of the latter identified upregulation of genes known to mediate resistance to BYL-719 (Le et al., 2016) and PI3K inhibition (Hanker et al., 2019). Expression of FOXM1 was largely unaffected by treatment with PI3Kα inhibitors in all six of these drug-resistant models. The clinical relevance of these observations was supported by analysis of RNA-seq data from paired tumor samples from patients that had been treated with BYL-719 (Le et al., 2016) and from immunohistochemical analysis of tumor biopsies from patients treated with tamoxifen and GDC-0032 (Baird et al., 2019). FOXM1 expression was unchanged or upregulated in BYL-719-resistant tumors compared with treatment naive tumors and was sustained in patients with progressive disease that had been treated with tamoxifen and GDC-0032.

Simultaneous blockade of PI3K and ER are needed for optimal treatment of ER^+^ breast tumors with aberrant activation of the PI3K pathway (Bosch et al., 2015). A phase II trial using taselisib (GDC-0032) and tamoxifen is currently underway (Baird et al., 2019), and the recently completed randomized phase III SOLAR-1 clinical trial demonstrated a clinically relevant benefit if endocrine therapy (fulvestrant) was combined with a PI3Kα inhibitor (BYL-719) in patients with HR^+^ HER2-advanced breast cancer with *PIK3CA* mutations (André et al., 2019). We have shown here that combining a PI3Kα inhibitor with tamoxifen or fulvestrant overcame acquired or engineered resistance to PI3Kα inhibition, resulting in decreased cell viability, marked decreases in FOXM1 and LDHA protein expression, and a decrease in hyperpolarized ^13^C label exchange between pyruvate and lactate.

The correlation observed here between persistent FOXM1 expression and resistance to PI3Kα inhibitors, which can be explained by phosphorylation of FOXO3a, suggested that FOXM1 may be involved directly in this drug resistance. The requirement for simultaneous blockade of both PI3K and ER in tumors with aberrant activation of the PI3K pathway (Bosch et al., 2015) supports this notion as both pathways converge on FOXM1. FOXM1 is an ERα transcriptional target that mediates its mitogenic functions and has been shown to play a role in breast cancer endocrine sensitivity and resistance (Millour et al., 2010). Elevated expression of FOXM1 predicts poor survival in ER^+^ and progesterone receptor-positive breast cancer and in patients treated with adjuvant chemotherapy or tamoxifen (Lu et al., 2018). Using the METABRIC dataset (Curtis et al., 2012), we showed that increased FOXM1 expression correlates with poorer survival in *PIK3CA* mutant breast cancer patients and that survival is decreased in ER^+^ breast cancer patients with FOXM1 copy number gain. Based on *FOXM1* gene expression in the primary tumor, risk of relapse is increased in ER^+^ breast cancer compared with ER^−^ breast cancer.

As well as demonstrating synergy between GDC-0032 and tamoxifen or fulvestrant treatment in tumors that were resistant to treatment with GDC-0032 alone, where the combined treatment lowered FOXM1, we have also demonstrated increased cell viability in drug-treated cells overexpressing FOXM1C and synergism between FOXM1 depletion and PI3Kα inhibition in tumor models with acquired or engineered resistance to PI3Kα inhibitors. These observations suggest that FOXM1 plays a direct role in drug resistance, which further supports its use as a biomarker of resistance to PI3Kα inhibition in ER^+^ *PIK3CA*-mutant breast cancer.

FOXM1 expression is elevated in many types of cancer, where it is involved in tumor initiation, progression, invasion, metastasis, angiogenesis, and drug resistance (Koo et al., 2012). Many of the mechanisms of resistance to PI3Kα inhibitors, including upregulation of *CDK4/6*, *KRAS*, or *PIK3CB* mutations; *FGFR1* amplification; and overexpression or aberrant activation of *RICTOR*, *PIM1*, *AXL*, *PDK-1/SGK1*, and *SGK3* (Hanker et al., 2019), will likely result in persistent FOXM1 expression following drug treatment. CDK4 and CDK6 activate FOXM1 (Anders et al., 2011), and FGFR1 appears to be involved in FOXM1 expression since silencing of FGFR1 in glioblastoma cells resulted in downregulation of FOXM1 (Gouaze-Andersson et al., 2018). Activation of RAS-ERK drives cell cycle progression by regulating the CDK2/Cyclin E and CDK1/Cyclin B complexes, which promote G1/S and G2/M transitions. These complexes also phosphorylate FOXM1, which is required for competency (Major et al., 2004). PIM-1 phosphorylates and thereby inhibits cell cycle inhibitors p21^CIP1/^WAF1 and p27^KIP1^, releasing their inhibitory effect on the CDK2/Cyclin E, CDK1/Cyclin B, and CDK4/6 complexes (Malinen et al., 2013), which in turn can phosphorylate and activate FOXM1. AXL dimerizes with epidermal growth factor receptor (EGFR) (Elkabets et al., 2015), which has been shown to regulate FOXM1 expression via an EGFR/RAS/FOXM1/β-catenin axis in colorectal cancer (Valverde et al., 2017). PDK-1 controls FOXM1 expression by phosphorylation of residue T308 of AKT, which is essential for full AKT activation (Yu and Cui, 2016), and PDK1/AKT have been shown to mediate FGF2-induced *FOXM1* expression in lung fibroblasts (Penke et al., 2018). The serine/threonine kinase SGK1 mediates phosphorylation of FOXO3a at Ser314, facilitating its nuclear export (Mori et al., 2014), and SGK-3 phosphorylates FOXO3a at multiple sites, preventing FOXO3a from localizing to the nucleus (Bruhn et al., 2013). In summary, these observations support the suggestion that FOXM1 may play a direct role in resistance to PI3Kα inhibition.

In conclusion, we have shown that FOXM1 expression is a biomarker of response and resistance to PI3Kα inhibition in ER^+^ *PIK3CA*-mutant breast tumors. Assessment of FOXM1 status would normally require a tissue biopsy; however, our data show that this can also be measured via its effects on LDHA expression using MRS of hyperpolarized [1-^13^C]pyruvate metabolism. Taken together, our findings have clinical implications for breast cancer and possibly other cancers where PI3K/FOXM1 signaling pathways are active.

## STAR★Methods

### Key Resources Table

**Table:** 

REAGENT or RESOURCE	SOURCE	IDENTIFIER
**Antibodies**		

Rabbit polyclonal anti-Phospho-Akt (S473)	Cell Signaling Technology	Cat#9271; RRID: AB_329825
Rabbit polyclonal anti-Akt	Cell Signaling Technology	Cat#9272; RRID: AB_329827
Rabbit polyclonal anti-Bactin	Abcam	Cat#ab8227; RRID: AB_2305186
Rabbit polyclonal anti-Histone H3 (CHIP)	Abcam	Cat#ab1791; RRID: AB_302613
Mouse monoclonal anti-HIF1-alpha	Abcam	Cat#ab1; [H1alpha67]; RRID: AB_296474
Rabbit polyclonal anti-HK-II	Cell Signaling Technology	Cat#2867; RRID: AB_2232946
Rabbit polyclonal anti-FOXO-3a	Cell Signaling Technology	Cat#2497; RRID: AB_836876
Rabbit monoclonal anti-FOXM1	Cell Signaling Technology	Cat#5436: RRID: AB_10692483
Rabbit polyclonal anti-FOXM1 (CHIP)	GeneTex	Cat#GTX100276; [C3]; RRID: AB_1240833
Rabbit polyclonal anti-FOXM1 (IHC)	Cell Signaling Technology	Cat#20459;[D3F2B]p; RRID: AB_2798842
Goat polyclonal anti-GLUT1	Santa Cruz Biotechnology	Cat#sc1603; [N-20]; RRID: AB_2254952
Goat polyclonal anti-GLUT3	Santa Cruz Biotechnology	Cat#sc31838; [I-14]; RRID: AB_2302128
Rabbit monoclonal anti-LDHA	Cell Signaling Technology	Cat#3582; [C4B5]; RRID: AB_2066887
Rabbit polyclonal anti-MCT1	Atlas	Cat#HPA003324; RRID: AB_1856982
Rabbit polyclonal anti-MCT4	Atlas	Cat#HPA021451; RRID: AB_1853663
Rabbit monoclonal anti-c-Myc	Abcam	Cat#Ab32072; [Y69]; RRID: AB_731658
Rabbit polyclonal anti-PTEN	Cell Signaling Technology	Cat#9552; RRID: http://antibodyregistry.org/AB_10694066
Rabbit polyclonal anti-Phospho-S6 (S235/236)	Cell Signaling Technology	Cat#2211; RRID: AB_331679
Rabbit monoclonal anti-P-S6 (IHC)	Cell Signaling Technology	Cat#4857; [91B2]; RRID: AB_2181035
Goat polyclonal anti-Rabbit Horseradish Peroxidase affiniPure HPR	Jackson ImmunoResearch	Cat#111-035-144; RRID: AB_2307391
Goat polyclonal anti-Mouse Horseradish Peroxidase secondary antibody affiniPure HPR	Jackson ImmunoResearch	Cat#115-035-062; RRID: AB_2338504
Donkey polyclonal anti-Goat Horseradish Peroxidase secondary antibody affiniPure HPR	Jackson ImmunoResearch	Cat#705-035-147; RRID: AB_2313587
Goat anti-rabbit secondary antibody Alexa Fluor 488	Life Technologies	Cat#A11034; AB_2576217
Mouse monoclonal PE anti-human CD298	Biolegend	Cat#341704; RRID: AB_2274458
Mouse monoclonal APC Class I (H-2Kd) Antibody	ThermoFisher Scientific	Cat#114613; RRID: AB_2750193

**Bacterial and Virus Strains**		

ONE SHOT STBL3 Competent Bacteria	ThermoFisher Scientific	Cat#C737303

**Biological Samples**		

Patient-derived xenografts (PDX) for explants	(Bruna et al., 2016)	HCI-001, HCI-011
Human breast tumor samples for IHC	Netherlands Cancer Institute Biobank (Baird et al., 2019)	N/A

**Chemicals, Peptides, and Recombinant Proteins**		

AZD-6482 (Astrazeneca), dissolved in DMSO	Selleckchem	Cat#S1462
BYL-719 (Novartis), dissolved in DMSO	Selleckchem	Cat#S2814
GDC-0032 (Genentech), dissolved in DMSO	Selleckchem/C.C. lab.	Cat#S7103
FX-11, dissolved in DMSO	Calbiochem	Cat#427218
FDI-6, dissolved in DMSO	Sigma	Cat#SML1392
4Hydroxitamoxifen, dissolved in EtOH	Sigma/C.C. lab.	Cat#SML1666
Tamoxifen, *in vivo* dissolved in 5%DMSO – Corn oil	Tocris Bioscience	Cat#6342
Fulvestrant, dissolved in DMSO, *in vivo* 5%DMSO – Corn oil	Selleckchem	Cat#S1191
Doxycycline hyclate, dissolved in H2O	Sigma	Cat#D9891
Alamarblue	ThermoFisher Scientific	Cat#DAL1025
FAST Syber green Master Mix	ThermoFisher Scientific	Cat#4385610
SuperScript II Reverse Transcriptase	ThermoFisher Scientific	Cat#18064014
Oligo(dT) 12-18 primer	ThermoFisher Scientific	Cat#18418012
Protease inhibitor cocktail	Roche	Cat#11836170001
17ß-Estradiol 0.72mg/pellet 90 Day Release	Innovative Research of America	Cat#NE-121
Proteinase K	ThermoFisher Scientific	Cat#26260
Dynabeads Portein A IP Kit	ThermoFisher Scientific	Cat#10006D
ProLong® Gold Antifade Reagent with DAPI	ThermoFisher Scientific	Cat#P36935
Matrix, Basement membrane; Corning; Matrigel; Growth Factor Reduced; Phenol Red-Free	BD Biosciences	Cat#356231
eBioscience™ Fixable Viability Dye eFluor™ 455UV	ThermoFisher Scientific	Cat#65-0868-14

**Critical Commercial Assays**		

Plasmid Maxi Kit	QIAGEN	Cat#12163
RNAeasy kit	QIAGEN	Cat#74104
Shredders	QIAGEN	Cat#79654
PCR Purification kit	QIAGEN	Cat#28104
Tumor Dissociation Kit, human	ThermoFisher Scientific	Cat#130-095-929
TruSeq Stranded mRNA high-throughput (HT) Sample Prep kit	Illumina	Cat# ID RS-122-2103

**Deposited Data**		

Raw sequencing data	This paper	The European Genome-phenome Archive at the European Bioinformatics Institute (EGEA), accesion number [EGAS00001004452]
Raw data	This paper	Apollo University of Cambridge Repositori: [https://doi.org/10.17863/CAM.55683; Mendeley: [https://data.mendeley.com/datasets/vjj4sdwhjf/draft?a=43464dd5-acb8-4e51-8d28-d3c540a6421a]
Analysis code for RNA sequencing data	This paper	http://github.com/cclab-brca/HCI011_RNAseq

**Experimental Models: Cell Lines**		

Human: MCF7	ATCC/CRUKCI Biorepository	Cat# HTB-22; RRID: CVCL_0031
Human: T47D	ATCC/CRUKCI Biorepository	Cat# HTB-133; RRID: CVCL_0553
Human: MDAMB231	ATCC/CRUKCI Biorepository	Cat# HTB-22; RRID: CVCL_0062
Human: MDAMB468	ATCC/CRUKCI Biorepository	Cat# HTB-26; RRID: CVCL_0419
Human: T47D PTEN KO	This paper	N/A

**Experimental Models: Organisms/Strains**		

Mouse: NSG: NOD.Cg*-Prkdc*^*scid*^*Il2rg*^*tm1Wjl*^*/*SzJ	Charles River	Cat# 614; RRID: IMSR_JAX:005557

**Oligonucleotides**		

PTEN_KD (shRNA PTEN; mirPTEN; ID#1523): 5′ TGCTGTTGACAGTG AGCGACCAGCTAAAGGTGAAGAT ATATAGTGAAGCCACAGATGTATA TATCTTCACCTTTAGCTGGCTGCC TACTGCCTCGGA	(Fellmann et al., 2013)	N/A
PTEN_KO: sgRNA TTATCCAAACATTATTGCTA	This paper	N/A
LDHA_KD: shRNA: 5′ CCGGCCAAAG ATTGTCTCTGGCAAACTCGAGTTT GCCAGAGACAATCTTTGGTTTTT	This paper	N/A
FOXM1_SEQ1_KD: 5′ CCGGGCCAA TCGTTCTCTGACAGAACTCGAGTT CTGTCAGAGAACGATTGGCTTTTT	This paper	N/A
FOXM1_SEQ1_KD: 5′ CCGGGCC CAACAGGAGTCTAATCAACTCGAGT TGATTAGACTCCTGTTGGGCTTTTTG	This paper	N/A
LDHA oligo1 and 2_ CHIP: 5′- TATCTCAAAGCTGCACTGGG and 5′- TGCTGATTCCATTGCCTAGC C	(Cui et al., 2014)	N/A
LDHA oligo3 and 4_ CHIP: 5′- CTGCAGGAAGCCATGATCA and 5′- TCCCACTCACAGTGAAGCCT	(Cui et al., 2014)	N/A
CCNB1 oligo1 and 2_CHIP: 5′- CGCGATCGCCCTGGAAACGCA and 5′- CCCAGCAGAAACCAACAGCCGT	(Gormally et al., 2014)	N/A

**Recombinant DNA**		

pcW57.1. FOXM1c	(Barger et al., 2015)	Addgene: #68810; RRID: Addgene_68810
TetOnPLKO.puro	(Wiederschain et al., 2009)	Addgene: #21915; RRID: Addgene_21915
pBOBI	Verma laboratory, Salk Institute La Jolla, USA	N/A, gift to K.M.B
TetOnPLKO.puro shRNA FOXM1 sequence 1	This paper	N/A
TetOnPLKO.puro shRNA FOXM1 sequence 2	This paper	N/A
TetOnPLKO.puro shRNA LDHA	This paper	N/A
pBOBI Strawberry_FOXM1C	This paper	N/A
pBOBI mirPTEN	This paper	N/A

**Software and Algorithms**		

MATLAB	MathWorks	http://www.mathworks.com/products/matlab/; RRID: SCR_001622
FIJI 2.0.0-rc-69/1.52p Image J	ImageJ	https://imagej.net/Fiji; RRID: SCR_002285 ImageJ
R	R Core Team	https://www.r-project.org/; RRID: SCR_001905
GraphPad Prism 5.0	GraphPad Prism	http://graphpad-prism.software.informer.com/5.0, RRID:SCR_002798
Vivoquant 3.0 software	Mediso USA	http://www.vivoquant.com/

### Resource Availability

#### Lead Contact

Requests for resources and reagents should be directed to and will be fulfilled by the Lead Contact, Kevin M. Brindle (kmb1001@cam.ac.uk).

#### Materials Availability

Materials generated in this study are available upon request.

#### Data and Code Availability

The RNA-seq dataset reported in this paper is available from the The European Genome-phenome Archive at the European Bioinformatics Institute (EGEA) with accession number: [EGAS00001004452]. The code supporting this study is available from: http://github.com/cclab-brca/HCI011_RNAseq. Original raw data have been deposited in Apollo – University of Cambridge Repository: https://doi.org/10.17863/CAM.55683 and in Mendeley Data: https://data.mendeley.com/datasets/vjj4sdwhjf/draft?a=43464dd5-acb8-4e51-8d28-d3c540a6421a.

### Experimental Model and Subject Details

#### Cell Lines

The cell lines generated in this study and their parental cell lines (T47D, MCF7, MDAMB231, MDAMB468) were grown in DMEM with 10% heat inactivated FBS. T47D and MCF7 cells were grown in media supplemented with non-charcoal stripped serum in order to preserve normal growth factor and hormone signaling. Cell lines were authenticated (STR profiling) and tested for mycoplasma contamination by Research Instrumentation and Cell Services (CRUK Cambridge Institute).

Drug concentrations: GDC-0032 (0.1 μM), BYL-719 (1 μM), AZD-6482 (1 μM), Tamoxifen (MCF7 1 μM or T47D 2 μM), Fulvestrant (1 μM), FDI-6 (5 μM), doxycycline (100 ng/ml) and FX-11 (10 μM).

#### Patient-Derived Tumor Cells (PDTCs), Disaggregated Cells from T47DR Tumors and PDXs

PDX models for tumor explants were previously published with all required ethical approvals (Bruna et al., 2016). PDTCs from HCI-001, HCI-011, HCI-011R tumors and cells from T47DR tumors were obtained by dissociating them using a kit (human 130-095-929, Miltenyi Biotec), as described previously (Bruna et al., 2016). Cells were grown in DMEM/F12 (1:1 ratio) media supplemented with B27 (GIBCO), basic EGF (20 ng ml-1, Sigma), FGF (10 ng ml-1, Sigma), Heparin (4 μg ml^-1^, Sigma) with penicillin-streptomycin (1%) and normocin (1%). Cell populations were analyzed for the presence of human breast tumor and mouse stromal cells using a Mouse Cell Depletion Kit (MiltenyiBiotec), as described in the Supplementary Methods Section (Flow cytometry of Human versus Mouse PDTC cultures).

#### Animals

NOD.Cg-Prkdcscid Il2rgtm1Wjl/SzJ female 6-8-week-old mice (NSG) were purchased from Charles River. All animal experiments were conducted in compliance with project and personal licenses issued under the Animals (Scientific Procedures) Act of 1986. Mice were housed according to, and protocols were approved by the Cancer Research UK, Cambridge Institute Animal Welfare and Ethical Review Body.

#### FFPE Clinical Samples

Clinical FFPE sections were obtained from the POSEIDON trial phase 1b, multi-center, dose-escalation study conducted in Amsterdam (the Netherlands), Barcelona (Spain) and Cambridge (UK) (Baird et al., 2019). The study was conducted in accordance with Good Clinical Practice and the Declaration of Helsinki and was approved by regulatory authorities, ethics committees and institutional review boards at each site. The use of archival human biospecimens and data for research purposes at the Netherlands Cancer Institute have been executed pursuant to Dutch legislation and international standards; International Guideline on Good Clinical Practice and the GDPR. Within this framework, patients are informed and have always had the opportunity to object or actively consent to the (continued) use of their personal data and biospecimens in research. Hence, the procedures comply both with international and national legislative and ethical standards. All patients had HR-positive breast cancer and provided written informed consent before taking part. POSEIDON is a European investigator-initiated trial, funded by the EU FP7 RATHER consortium (project ID: 258967) and EurocanPlatform (project ID: 260791), with additional support from an unrestricted research grant from Genentech, and led by the Netherlands Cancer Institute (Amsterdam, the Netherlands), Cambridge Cancer Center (Cambridge, UK), and Vall d'Hebron Institute of Oncology (Barcelona, Spain). The investigators would like to thank the patients who took part in the study, and their families. Support is also acknowledged from the NKI Data Center, the NKI Core Facility Molecular Pathology and Biobanking (CFMPB), Cancer Research UK Cambridge Cancer Centre, and the Cambridge: Experimental Cancer Medicine Center (ECMC); Cancer Molecular Diagnostics Laboratory (CMDL); NIHR Biomedical Research Center (BRC); and Cambridge Clinical Research Center (CCRC).

### Method Details

#### Cell Lines

##### PTEN KD

Lentiviral vectors expressing an shRNA PTEN or shRNA control-mir based cassette and mStrawberry (Fellmann et al., 2013) were constructed by assembling the transgenes into the lentiviral vector pBOBI (Miyoshi et al., 1998). *PIK3CA* mutant cell lines MCF7 (E545K) and T47D (H1047R) were transduced and Strawberry-positive cells sorted using a BD FACSAria cell sorter. The hairpin sequences were: Ctrl (Renilla luciferase) 5′ TGCTGTTGACAGTGAGCGCAGGAATTATAATGCTTATCTATAGTGAAGCCACAGATGTATAGATAAGCATTATAATTCCTATGCCTACTGCCTCGGA and mirPTEN 5′ TGCTGTTGACAGTGAGCGACCAGCTAAAGGTGAAGATATATAGTGAAGCCACAGATGTATATATCTTCACCTTTAGCTGGCTGCCTACTGCCTCGGA.

##### PTEN KO

For PTEN knockout, a guide targeting human PTEN (TTATCCAAACATTATTGCTA) was cloned into pX459 (Addgene). This was transfected into T47D cells using lipofectamine 3000, which were then puromycin-selected.

sgPTEN forward 5′-

CACCGTTATCCAAACATTATTGCTA

sgPTEN reverse 5′-

AAACTAGCAATAATGTTTGGATAAC

##### LDHA-KD, FOXM1-KD and FOXM1C-mStrawberry

shRNA sequences targeting human LDHA and FOXM1 were cloned into the TetOnPLKO lentiviral vector (Wee et al., 2008; Wiederschain et al., 2009). The human FOXM1c open reading frame was subcloned from the FOXM1c lentiviral vector pcW57.1 (Addgene, plasmid 68810 gift from Adam Karpf, (Barger et al., 2015)) into a pBOBI plasmid (gift from Verma laboratory, Salk Institute La Jolla, USA). The plasmid contains an EF1 promoter used to drive transcription of the red fluorescent protein, mStrawberry, and FOXM1C. The mStrawberry coding sequence was separated from the FOXM1C coding sequence by an E2A sequence, which resulted in the co-expression of mStrawberry and FOXM1C in equimolar concentrations from a single mRNA transcript (Szymczak-Workman et al., 2012). Lentiviruses were produced in HEK 293T cells with the shRNA and packaging plasmids (pCMVΔR8.91 (gag-pol) and pMD.G (VSV-G glycoprotein)), or pBOBI and packaging plasmids pMDL (gag-pol), pRev (Rev) and pVSV-G (VSVG). Lentiviruses were collected 48 h after transfection, mixed with polybrene (8 μg/ml) and used for infection. For shRNA experiments, cells were selected in puromycin (2 μg/ml) for at least 48 h and for cells expressing mStrawberry cells displaying similar red fluorescence were sorted after 72 h, using a BD FACSAria cell sorter.

shLDHA forward 5’-

CCGGCCAAAGATTGTCTCTGGCAAACTCGAGTTTGCCAGAGACAATCTTTGGTTTTT

shLDHA reverse 5′-

AATTAAAAACCAAAGATTGTCTCTGGCAAACTCGAGTTTGCCAGAGACAATCTTTGGC

shFOXM seq1 forward 5′-

CCGGGCCAATCGTTCTCTGACAGAACTCGAGTTCTGTCAGAGAACGATTGGCTTTTT

shFOXM seq1 reverse 5′-

AATTAAAAAGCCAATCGTTCTCTGACAGAACTCGAGTTCTGTCAGAGAACGATTGGC

shFOXM1 seq2 forward 5′-

CCGGGCCCAACAGGAGTCTAATCAACTCGAGTTGATTAGACTCCTGTTGGGCTTTTTG

shFOXM1 seq2 reverse 5′-

AATTCAAAAAGCCCAACAGGAGTCTAATCAACTCGAGTTGATTAGACTCCTGTTGGGC

shCtrl forward 5′-5′CCGGCCTAAGGTTAAGTCGCCCTCGCTCGAGCGAGGGCGACTTAACCTT AGG

shCtrl reverse 5′-5′AATTCCTAAGGTTAAGTCGCCCTCGCTCGAGCGAGGGCGACTTAACCTT AGG

#### Xenografts

Fragments of HCI-001 and HCI-011 (Bruna et al., 2016) xenografts were implanted with Matrigel subcutaneously in 6-8-week-old female NSG mice (NOD.Cg-Prkdcscid Il2rgtm1Wjl/SzJ, Charles River). The population dynamics of genomically-defined clones are unaffected by the site of tumor implantation: subcutaneous, under the renal capsule or in the mammary fat pad (Eirew et al., 2015) and the treatment responses of breast cancer PDXs implanted subcutaneously have been shown to be similar to those found in the clinic (Yu et al., 2017).

T47D breast cancer cell lines were implanted subcutaneously in 6-week-old female NSG mice together with a subcutaneous estrogen pellet (0.72 mg/pellet 90-day release; Innovative Research of America, NE-121).

Tumor volumes were assessed by caliper measurements, using the ellipsoidal volume formula: 1/2 x (length x width^2^). When the tumors reached a volume of ∼200-300 mm^3^ (for prolonged drug treatment experiments) or ∼500-1000 mm^3^ (for imaging), mice were randomly assigned to groups that received:

A) Prolonged treatment: drug vehicle or GDC-0032 were administered orally once a day (10 mg/kg; 5 days on/2 days off). Tamoxifen was administered intraperitoneally (i.p.) (20 mg/kg, 5 days on/2 days off).
B) Short term treatment: drug vehicle or GDC-0032 were administered orally once a day (10 mg/kg; 3 doses). Tamoxifen was administered i.p. (20 mg/kg; 3 doses) and Fulvestrant subcutaneously (25 mg/kg, 1 dose)

GDC-0032 was dissolved in 0.5% methylcellulose/0.2% Tween-80. Tamoxifen (8.8 mg/ml) was dissolved in sterile filtered corn oil. Fulvestrant (10 mg/ml) was dissolved in sterile filtered corn oil.

For induction of shRNA expression, animals received a standard or doxycycline diet *ad libitum* (0.2 g doxycycline/kg food pellets, Harlan D.98186).

#### ^13^C Magnetic Resonance Spectroscopy

Mice were anesthetized with 1-2% isoflurane (Isoflo, Abbotts Laboratories Ltd, Maidenhead, UK) in air/O_2_ (75/25% vol/vol, 2 L/min). Body temperature was maintained with warm air. [1-^13^C]pyruvic acid samples (44 mg, 14 mol/L; 99% ^13^C) containing 15 mmol/L of trityl radical, tris (8-carboxy-2,2,6,6-tetra-(hydroxyethyl)-benzo-[1,2-4,50]-bis-(1,3)-dithiole-4-yl)-methyl sodium salt (OX063; GE Healthcare, Amersham, UK) and 1.5 mmol/L of an aqueous solution of a gadolinium chelate (Dotarem, Guerbet, Roissy, France) were hyperpolarized using a 3.35-T Oxford Instruments Hypersense polarizer (Oxford Instruments, Abingdon, UK) and dissolved at 180°C in 6 mL buffer containing 40 mM HEPES, 94 mM NaOH, 30 mM NaCl and 50 mg/L EDTA.

Spectra were acquired at 7.0-T (Agilent, Palo Alto, CA) using a ^13^C/^1^H volume transmit coil (Rapid Biomedical, Rimpar, Germany) with a 20-mm diameter ^13^C receiver coil (Rapid Biomedical). Hyperpolarized [1-^13^C]pyruvate (0.3 mL, 82 mM) was injected i.v. via a tail vein catheter and spectra acquired using a slice selective pulse, with a nominal flip angle of 5°, a slice thickness of 6-10 mm, 6010 Hz bandwidth and 1024 data points. Spectra were acquired every second for 3 minutes. Data were processed in Matlab (Mathworks, Natick, USA).

Three dimensional single-shot, images of [1-^13^C] pyruvate and [1-^13^C] lactate were acquired as a time course, using the method described in (Wang et al., 2017). Images of each metabolite were acquired every 2 seconds in an interleaved manner with pyruvate image acquisition beginning 2 s after the start of hyperpolarized pyruvate injection and lactate image acquisition commencing after 11 seconds. Hyperpolarized ^13^C-labelled metabolite false-color images are presented as an overlay on T_2_-weighted ^1^H images (256x256 data points, 3.6 or 4.2 cm field-of-view, 8 echoes with an effective echo time of 50 ms and a TR of 1.8 s), with the lactate signal scaled to be ten times that of the pyruvate signal. The central 2 slices of the metabolite images (covering 5 mm through the center of the tumor) were summed as were the first 16 seconds of acquisition for each metabolite.

#### [^18^F]FDG PET/CT

Mice were fasted for 4-8 hours prior to induction of anesthesia with 1 – 2.5% isoflurane. 2-deoxy-2-[^18^F]fluorodeoxyglucose (12.1±1.1 MBq) (Alliance Medical, Guildford, UK) was injected intravenously *via* a tail vein. Data were acquired between 90- and 110-min post-injection in list-mode format on a NanoPET/CT scanner (Mediso, Hungary). Static PET images with a nominal isotropic resolution of 0.4 mm were reconstructed using a three-dimensional ordered-subset expectation maximization method with one to three coincidence modes, four iterations and six subsets. Images were normalized and corrected for decay, dead-time, random events and attenuation. For anatomical reference and attenuation correction a whole-body helical CT was acquired. The images were analyzed using Vivoquant 3.0 software (InviCRO, Massachusetts, USA). A region of interest was drawn manually over the subcutaneous tumor and Otsu thresholding applied to delineate the tumor.

#### RNA Extraction for RT–qPCR and RNA-seq

Total RNA was isolated using a RNeasy kit (Qiagen, Hilden, Germany). Flash-frozen tumor fragments were placed in RLT buffer and homogenized using a Tissue Lyser II (Qiagen) at a frequency of 30 s^-1^ for 30 seconds, centrifuged for 2 min at 16000g to pellet debris and RNA isolation was performed following the kit instructions.

For RT-qPCR: RNA was used for first strand cDNA synthesis with SuperScript II Reverse Transcriptase and oligo-dT primers (ThermoFisher Scientific - Invitrogen, Carlsbad, CA, USA). Reactions were performed in triplicate. Relative mRNA content was calculated using the comparative CT method after normalization to the Geomean of two housekeeping genes, β-actin (Bactin) and β_2_-microglobulin (B2M). The primers from Qiagen are: Hs Bactin QT00013209; Hs B2M QT00088935; Hs HK-II QT00095431; Hs FOXM1 QT00000140; Hs LDHA QT00001687; and Hs PTEN QT00086933 (Hs: Homo sapiens).

For RNAseq libraries for Illumina sequencing were prepared using TruSeq Stranded mRNA high-throughput (HT) Sample Prep kit (Cat ID RS-122-2103, Illumina) according to the manufacturer’s instructions. An input of 500 ng of total RNA was used for library preparation. After 12 cycles of PCR used in the Enrichment of DNA Fragments step, all libraries were quantified using KAPA Library Quantification Kit Illumina ROX Low (Cat ID, KK4873, KAPA Biosystems) and normalized to 10 nΜ. Libraries were then pooled in equal volumes and the pools were used for clustering on the HiSeq4000 sequencing platform (Illumina), according to the manufacturer's instructions. Sequencing was performed using 50 bp single-end (SE) reads to generate on average 10 million total reads per library.

Prior to alignment, sequencing quality was enforced using Trim Galore! (v0.4.2; http://www.bioinformatics.babraham.ac.uk/projects/trim_galore/). Then, as described in (Callari et al., 2018), reads were aligned to a combined human (hg19) and mouse (mm10) reference genome using STAR (v2.5.2b) (Casper et al., 2018). Counts were assigned to genes using featureCounts (v1.5.2), whereby the alignment score is used to distinguish reads as being sourced from human or mouse (Liao et al., 2014). Counts were then analyzed using the edgeR and limma packages (McCarthy et al., 2012; Ritchie et al., 2015; Robinson et al., 2010). Codes for Figures are accessible at url (http://github.com/cclab-brca/HCI011_RNAseq).

#### Western Blots

Cells or tumors were flash-frozen in liquid nitrogen, then harvested in RIPA buffer (Pierce, 89901) with complete mini EDTA-free inhibitor cocktail (Sigma, 4693159001). Tumor samples were homogenized using a Precellys Homogenizer (Bertin Instruments). Whole cell extracts were sonicated using a Bioruptor Plus (Diagenode) for 2 min (30 sec on/30 sec off) to degrade DNA, and centrifuged for 5 min at 16000g to remove debris. Protein concentration in the supernatant was quantified by a Direct Detect Spectrometer (Millipore) and samples then run on NuPAGE 4-12% bis-Tris gels (Invitrogen). Proteins were transferred to nitrocellulose membranes using an iBlot2 (Invitrogen) or by wet transfer (BioRad) and the membrane was then blocked with 3% bovine serum albumin or 5% non-fat dried milk, and then incubated with antibodies, which were detected using ECL Prime-reagent (Amersham). Actin was used as a loading control. Western blot densitometry was performed using Fiji (ImageJ) image analysis software (Schindelin et al., 2012). The numbers beneath the blots represent the band intensities normalized to band intensities of vehicle-treated controls.

#### Immunofluorescence

Cells seeded in 35 mm dishes at 0.1 x 10^6^ cells/ml were fixed with 4% paraformaldehyde, permeabilized using 1% Triton X-100 in 3% BSA in PBS and incubated with anti-FOXO3a antibody (1:500) in 1% BSA for 1 hour, and stained with Alexa Fluor 488–conjugated anti-rabbit IgG antibody. The cells were mounted with ProLong® Gold Antifade Reagent and with DAPI (ThermoFisher Scientific). Images were captured using a Leica TCS SP5 confocal microscope (Leica Microsystems).

#### Cell Viability Assays

Cells seeded in 96 well format plates at 0.01 x 10^6^ cells/ml were analyzed after drug treatment for 120 h. Cells were treated with 10% Alamarblue (ThermoFisher), followed by 1–4 h incubation at 37°C, and for PDTCs overnight at 37°C. Experiments were performed in four or five technical replicates and three to four biological replicates. Fluorescence was excited at 570 nm (ClarioStar, BMG LABTECH).

#### Flow Cytometric Measurements of mStrawberry Fluorescence

A mixture of parental MCF7 cells and MCF7 cells co-expressing mStrawberry and FOXM1C or parental T47D cells and T47D cells co-expressing mStrawberry and FOXM1C were trypsinized following treatment with drug vehicle or GDC-0032 for 120 h. After a PBS wash, the samples were resuspended in 0.5 ml PBS and kept at 4°C before flow cytometric measurements of mStrawberry fluorescence (MACSQuant Analyzer, Miltenyi Biotec, Bergisch Gladbach, Germany). The fraction of cells expressing mStrawberry in the drug-treated relative to the vehicle-treated cell populations was measured after counting 10,000 events.

#### [^18^F]FDG Uptake

After 72 h of drug or vehicle treatment, one ml of glucose-free medium containing [^18^F]FDG (37 KBq/ml) was incubated with 1 x 10^6^ cells for 30 min. The cells were then washed with PBS and lysed in RIPA buffer (Pierce, ref 89900). [^18^F]FDG content was measured using a well counter. Experiments were performed in three biological replicates and counts normalized to protein content.

#### LDH Activity

After 72 h of drug or vehicle treatment, LDH activity was determined by measuring the initial rate of pyruvate reduction and is expressed in units per milligram of protein, where 1 unit is defined as the amount of enzyme that oxidizes 1 μmol of NADH in 1 min at 37°C, pH 7.1. The reduction in NADH absorbance at 340 nm was monitored using a PheraStar plate analyzer (BMG LABTECH). Assays were performed in three biological replicates (except for Figure 1C).

#### Lactate Measurements

Metabolites from snap frozen tissue (21 to 57 mg) were extracted with 2 M PCA, and 50 nmol TSP was added for use as a ^1^H NMR reference. Tissue was disrupted using a Precellys Tissue Homogenizer (Bertin Instruments). The sample pH was adjusted to 7.0 before being snap frozen, freeze dried and then dissolved in ^2^H_2_O. ^1^H spectra were acquired using a 600 MHz Avance III NMR-spectrometer (Bruker, Karlsruhe, Germany) with a BBI probe using a pulse-acquire sequence with water presaturation, a 90-degree excitation pulse and 5 s repetition time. Spectra were acquired into 8192 complex points, zero-filled to 16384 points, line broadened with a 3Hz Gaussian line shape and Fourier transformed. They were phased and referenced to the major TSP peak at 0 ppm and baseline corrected and the methyl proton peaks of lactate (doublet between 1.35ppm-1.31ppm) and TSP (singlet between 0.02ppm to -0.02ppm) were integrated. After correction for proton number (3 and 9 respectively) the ratio of peak amplitudes was used to calculate the lactate concentration.

#### Measurements of Glycolytic Flux

An eXF96 Extracellular Flux Analyzer (Seahorse Bioscience, North Billerica, MA, USA) was used to determine the effects of the drugs on lactate production (culture medium acidification) in T47 Ctrl and PTEN KO cells. Cells were seeded at 40,000 cells/well into 96-well Seahorse culture plates and treated with or without drugs for 72 h. Cells were then incubated for 1 hour at 37°C after replacement of the medium with Seahorse incubation medium containing 10 mM glucose, 2 mM L-glutamine and 5 mM HEPES (pH 7.4), with or without drugs. Incubations were performed in a CO_2_-free incubator to ensure accurate measurements of extracellular pH. Measurements of total extracellular acidification rate (ECAR, sum of glycolytic acidification, in the form of lactate^-^ plus H^+^ and the respiratory acidification, in the form of CO_2_ (which hydrates to H_2_CO_3_ then dissociates to HCO_3_^-^ plus H^+^)) were performed according to the manufacturer’s instructions. ECAR is presented as mean ± standard deviation (S.D.) of experimental quintuplicates.

#### FOXM1 CHIP

FOXM1 binding sites in the LDHA promoter have been described previously (Cui et al., 2014). Cells were washed with phosphate-buffered saline (PBS) and crosslinked with 1% formaldehyde for 10 min at 37 °C. The reaction was stopped by the addition of glycine to 125 mM final concentration. Samples were sonicated to generate DNA fragments with an average size below 1000 base pairs, followed by immunoprecipitation with the indicated antibodies. Bound DNA fragments were eluted and amplified by PCR using the following primer pairs: LDHA oligo 1 5’- TATCTCAAAGCTGCACTGGGC; LDHA oligo 2 5′- TGCTGATTCCATTGCCTAGC; LDHA oligo 3 5′- CTGCAGGAAGCCATGATCA; LDHA oligo 4 5′- TCCCACTCACAGTGAAGCCT; CCNB1 oligo 1 5′- CGCGATCGCCCTGGAAACGCA; CCNB1 oligo 2 5′- CCCAGCAGAAACCAACAGCCGT.

#### Flow Cytometry Analysis of Human and Mouse Cells in PDTC Cultures

We used the human-specific antibody CD298 (PE; BioLegend, cat. no. 341704) and the mouse-specific antibody H-2Kb/H-2Db (APC; Thermo Fisher Scientific, cat. no. 114613). Flow cytometry was performed using a BD FACSymphony analyzer. Cell viability was determined by negative staining with eBioscience™ Fixable Viability Dye eFluor™ 455UV (Thermo Fisher Scientific, 65-0868-14). The forward-scatter area by forward-scatter width (FSC-H × FSC-A) and side-scatter area by side-scatter width (SSC-H × SSC-A) were used to discriminate single cells from doublet and multiplet cells. The following voltage settings were used: Size (488/FSC/290); Granularity (488/SSC/200); UV455_VD (355/431-28/330); APC-mMHCl (638/586-15/430); PE-CD298 (561/670-30/430).

#### Immunohistochemistry of Prelinical and Clinical Samples

Sections of formalin-fixed paraffin-embedded tissue (FFPE, 3 μm for preclinical samples) were stained with different antibodies (see reagent section). Slides were scanned at 20x magnification with a resolution of 0.5 μm per pixel on an Aperio AT2 (Leica Biosystems). Tumor sections were run on Leica’s Polymer Refine Detection System (DS9800) using a modified version of their standard template on the automated Bond-III platform. The de-waxing and re-hydration (as standard) prior to IHC used an automated Leica ST5020, as did the post-IHC de-hydration and clearing. Antigen retrieval was performed by incubating the slides with Tris-EDTA buffer at 100°C for 20 minutes. An anti-rabbit poly HRP-IgG containing 10% (v/v) animal serum in Tris-buffered saline/0.09% ProClin™ 950 was used. ProClin™ 950 was used as secondary antibody, 0.1% hematoxylin as counterstain and DAB Enhancer (Leica, AR9432) was used to intensify signals. Sections were mounted on a Leica coverslipper CV5030.

#### FISH Analysis of HCI-001 and HCI011 PDX FFPE Tumor Sections

Simultaneous detection of human and mouse centromeres was performed on FFPE sections. Briefly, 3 μM thick sections were cut, baked for 1 hour at 60°C before deparaffinization in xylene and rehydration through graded ethanol. Pre-treatments using Aquarius Tissue Pretreatment Kit (Cat # LPS 100, Cytocell) were carried out for 10 min at 98°C using pretreatment solution 1, followed by 8 min at 37°C using pretreatment solution 2, according to the manufacturer's instructions. Slides were dehydrated through graded ethanol. Ready-to-use All Human Centromere probe (Cat # KBI20000R, Leica Biosystems) and ready-to-use Mouse Pan Centromeric probe (Cat # 1697-MF-01, Cambio) were applied to the same slide and coverslips were sealed with Fixogum rubber cement (Cat # ICNA11FIXO0125, VWR). Slides were denatured for 5 min at 75°C before hybridization overnight in a humid chamber at 37°C. Coverslips were removed and slides were washed in 0.4X SSC buffer for 2 min at 72°C, before a brief wash in 2X SSC plus 0.05% Tween 20. Slides were incubated with DAPI counterstain (Cat # 4083S, Cell Signaling Technologies), diluted to 10 μg/ml, for 5 min at room temperature in the dark. Slides were washed in ultrapure water and mounted with Prolong Diamond (Cat # P36970, ThermoFisher Scientific). The slides were imaged on the AxioScan (Zeiss) to create whole slide images. Images were captured at 40x magnification, with a resolution of 0.25 μm per pixel.

#### MSKCC Patient RNAseq Data Re-analysis

Patient tumor samples were obtained under Memorial Sloan Kettering Cancer Center (MSKCC) protocol #12-245 (clinicaltrials.gov ID: NCT01775072) approved by the MSKCC Institutional Review Board (IRB), and all participating patients provided written informed consent. The studies were conducted in accordance with the Declaration of Helsinki. Total RNA was extracted from formalin-fixed, paraffin-embedded (FFPE) tumor specimens using AllPrep DNA/RNA Kit (QIAGEN) according to the manufacturer's instructions. Total RNA was assessed for quality using the Caliper LabChip GX2. The percentage of fragments with a size greater than 200 nucleotides (DV200) was calculated using Illumina Fragment Analyzer. An aliquot of 200 ng of RNA was used as the input for first strand cDNA synthesis using Illumina's TruSeq RNA Access Library Prep Kit. Synthesis of the second strand of cDNA was followed by indexed adapter ligation. Subsequent PCR amplification enriched for adapted fragments. The amplified libraries were quantified using an automated PicoGreen assay. 200 ng of each cDNA library, not including controls, were combined into 4-plex pools. Capture probes that target the exome were added and hybridized for the recommended time. Following hybridization, streptavidin magnetic beads were used to capture the library-bound probes from the previous step. Two wash steps effectively remove any non-specifically bound products. These same hybridization, capture and wash steps are repeated to assure high specificity. A second round of amplification enriches the captured libraries. After enrichment the libraries were quantified with qPCR using the KAPA Library Quantification Kit for Illumina Sequencing Platforms and then pooled equimolarly. Pooled libraries were normalized to 2 nM and denatured using 0.1 N NaOH prior to sequencing. Flowcell cluster amplification and sequencing were performed according to the manufacturer's protocols using HiSeq 2500. Each run was a 76 bp paired-end with an eight-base index barcode read. Data were analyzed using the Broad Picard Pipeline, which includes de-multiplexing and data aggregation.

#### METABRIC Follow-Up Data Re-analysis

Expression of *FOXM1* and *PTEN* genes was normalized, as described in (Curtis et al., 2012). Expression log intensities were standardized using z-scores. Follow-up data from METABRIC was curated and processed as described in (Rueda et al., 2019). Figures S6B and S6C were computed using Kaplan-Meier estimates for disease-specific survival according to expression of FOXM1 split into three groups according to 15% and 85% percentiles. For Figure S5D a multistate model for breast cancer progression was fitted as described in (Rueda et al., 2019). The relapse hazard ratio for ER^-^ and ER^+^ disease for relapse after surgery, loco-regional or distant relapse and the hazard ratio for disease-specific death according to FOXM1 expression in the primary tumor were computed together with a 95% confidence interval. Other covariates in the Cox model include age, tumor grade, tumor size, number of positive lymph nodes and time since relapse.

### Quantification and Statistical Analysis

Graphs were generated using GraphPad Prism 5.0 (GraphPad software). Experiments were performed with at least three biologically independent replicates, unless stated otherwise. The list of tests used to determine statistical significance are included below. Statistical significance was defined as a p value of equal to or less than 0.05. If displayed as symbols, p values are depicted as: ^∗^p≤0.05 and ^∗∗^p≤0.005. n.s. = not significant.

One-sample Student's t-tests were considered when testing the location of a single mean (Figures S2A and S4G). Normality was assumed on the linear (i.e., non-transformed) scale and tests were performed on the average of the 3 (Figure S2A) or 5 (Figure S4G) technical replicates per biological replicate.

Paired Student's t-tests were considered when comparing the means of two groups under a paired design and assuming normality of the paired differences on the linear scale (Figures 1F, 1H, 2D, and 2F).

Welch's t-tests were considered when comparing the means of independent samples, assuming normality on the linear scale (Figures 1C, 1G, 1I, 2B, 2E, 3E, 3F, 4F–4I, 6E, 7B, 7J, S1A, S2E–S2G, S3C, S4A, and S4E). In analyses involving technical replicates, Welch's t-tests were performed on the average of the 2 (Figure S2D), 3 (Figures 4C, 7E, 7G, S3D, S3F, S3B, S5D, and S5E), 4 (Figures 3C, 7C, S2C, and S3A) or 5 (Figures 3A, 7D, 7F, S3E, S3G, and S3C) technical replicates. All tests of locations were two-sided and performed by means of the function T.TEST of Microsoft Excel [version 16.37] (with relevant options for the one- or two-sample case, and for the paired versus independent scenario) and controlled by means of the function t.test() in R [version 4.0.0].

Analyses of the evolution of cell viability per concentration and of the evolution of tumor size as a function of time were performed by means of linear mixed models, allowing modelling of within-experiment and within-mouse dependence by means of random effects. They were fitted via restricted maximum likelihood in R [version 4.0.0] by means of the function lme() of the package nlme [version 3.1-147]. Random intercept and slope models were considered in most cases (Figures 2D, 2A, 3D, 6B, 6B, 6D, and 7I). Random intercept only models were considered when more complex models could not be fitted or when model checks and likelihood ratio tests show no advantage in considering more complex models (Figures 1A, 6H, and 7I). To tackle heteroscedasticity, data were often fitted on a transformed scale (Figures: cubic root for 1A, 2D, 2A, 6B, 6H, and 7I) and/or the variance of the residuals was allowed to vary per group (Figures 2D, 2A, 3A, and 6D) or time (Figure 6B). Pointwise linear mixed models were fitted when modelling changes of growth at different time points (Figures 2D, 6H, and 7I).

All tests of the contrasts of interest were two-sided.

#### Survival Analyses

Survival curves were displayed by means of Kaplan-Meier plots (Figures S6B–S6D) and log-rank tests, performed with the function survdiff() of the package survival [version 3.1-12] in R [version 4.0.0], were used to compare the survival curves through time (Figures S6B–S6D). Confidence intervals for log hazard ratios were based on the estimates and standard errors of Cox regression fits obtained by means of the function coxph() of the R survival package.

## Supplemental Information

Document S1. Figures S1–S6

Document S2. Article plus Supplemental Information
