# Supplementary material for: Metabolic Imaging Detects Resistance to PI3Kα Inhibition Mediated by Persistent FOXM1 Expression in ER+ Breast Cancer
Source: Cancer Cell. 2020 Oct 12;38(4):516–533.e9. doi: 10.1016/j.ccell.2020.08.016 (PMC7562820; doi:10.1016/j.ccell.2020.08.016)
Supplement: Document S1. Figures S1–S6 [file mmc1.pdf]

Cancer Cell, Volume *38*

Supplemental Information

Metabolic Imaging Detects Resistance to PI3Kα Inhibition Mediated by Persistent FOXM1 Expression in ER^+ Breast Cancer

Susana Ros, Alan J. Wright, Paula D'Santos, De-en Hu, Richard L. Hesketh, Yaniv Lubling, Dimitra Georgopoulou, Giulia Lerda, Dominique-Laurent Couturier, Pedram Razavi, Rapahel Pelossof, Ankita S. Batra, Elizabeth Mannion, David Y. Lewis, Alistair Martin, Richard D. Baird, Mafalda Oliveira, Leonora W. de Boo, Sabine C. Linn, Maurizio Scaltriti, Oscar M. Rueda, Alejandra Bruna, Carlos Caldas, and Kevin M. Brindle

Supplemental figures

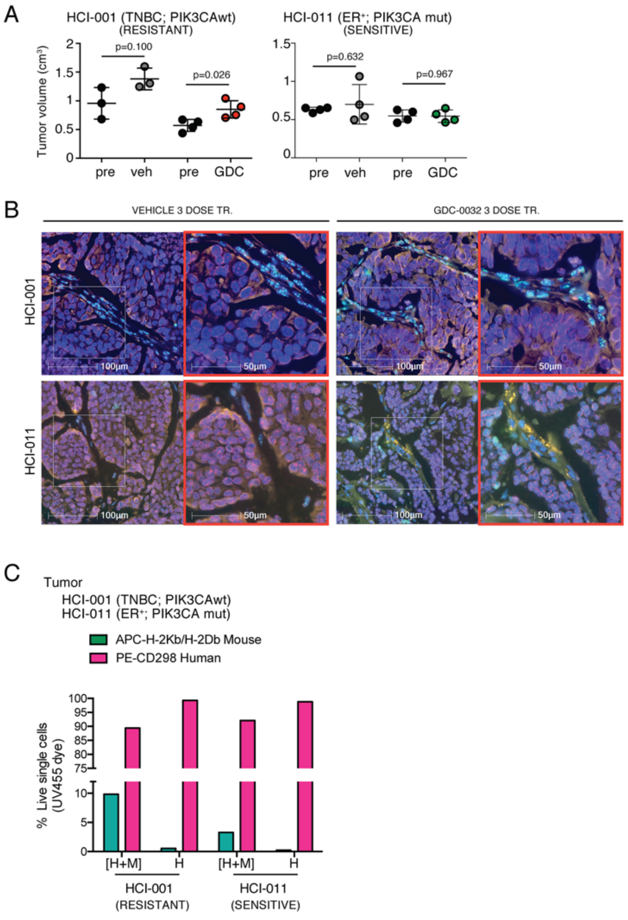

Figure S1, related to Figure 1. Resistance to a PI3Kα inhibitor can be detected using metabolic imaging with hyperpolarized [1-^{13}C]pyruvate

A) HCI-001 and HCI-011 tumor volumes (cm^3) before (pre) and after short-term treatment with GDC-0032 (HCI-001 n=4; HCI-011 n=4) or drug vehicle (HCI-001 n=3; HCI-011 n=4). P-values were calculated using two-sided Welch's T-tests.

B) FFPE sections of HCI-001 and HCI-011 tumors treated short-term with vehicle (left) or GDC-0032 (right) were stained for simultaneous detection of human (red) and mouse (green) centromeres (FISH analysis); with a DAPI counterstain (blue). Images were captured at 40x magnification.

C) Percentage of human (PE-CD298 positive) and mouse (APC-H-2Kb/H-2Db positive) live cells isolated from untreated disaggregated tumors determined by flow cytometry using a BD FACSymphony analyzer. Percentages of human breast cancer epithelial and mouse stomal cells (H+M), or human breast cancer epithelial cells (H) are shown. Mean of 1 biological experiment. Live cells were determined by dye-staining, eBioscience^{TM} Fixable Viability Dye eFluor^{TM} 455 nm negative.

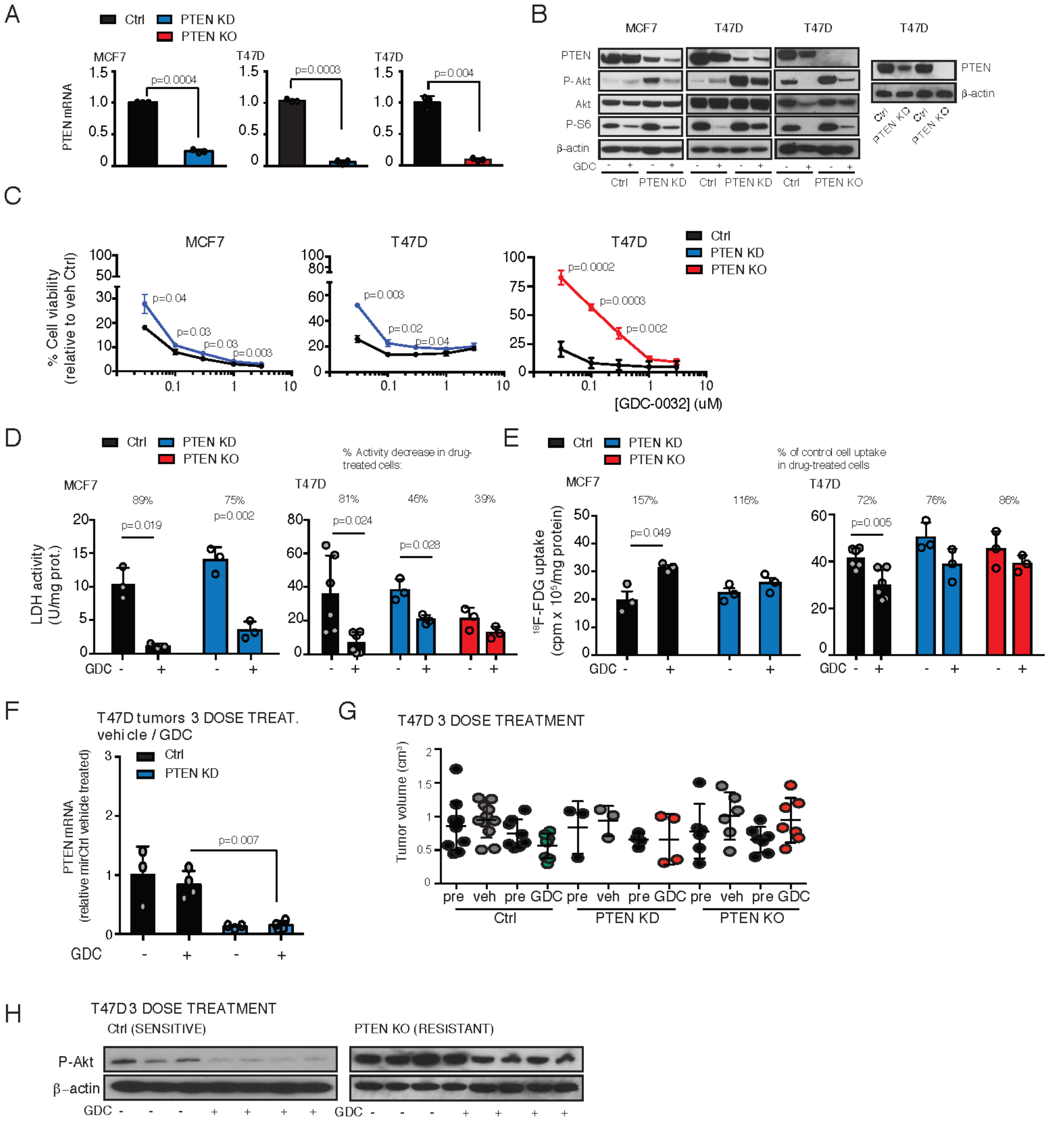

Figure S2, related to Figure 2. Imaging with hyperpolarized [1-^{13}C]pyruvate detects sensitivity and induced resistance to PI3Kα inhibition

We engineered MCF7 or T47D cells to knockdown or knock out PTEN expression using a synthetic shRNA embedded in a miR30 backbone (PTEN KD) or by using CRISPR-Cas9 (PTEN KO).

A) Cells were seeded at low density and expression of *PTEN* was determined by qPCR and normalized by the geomean of the housekeeping genes *β-ACTIN* and *B2M*. Figure shows the mean ± SD per condition (n=3). P-values were calculated using one-sided T-tests on the averages of technical replicates.

B) Expression of PI3K/Akt/mTOR pathway proteins in cells used in A) treated with drug vehicle or GDC-0032.

C) Viability of cells used in A) treated with different concentrations of GDC-0032 for 120 h, starting 24 h after plating. Figure shows the mean ± SD per group and drug concentration (n=3, 5 technical replicates). P-values were calculated using two-sided Welch's T-tests on the averages of technical replicates.

D) LDH activity measured in cells used in A) treated with drug vehicle or GDC-0032 for 72 h, starting at 24 h after plating. Figure shows the mean ± SD (n=3 to 6 experiments). The figures show the % decrease in LDH activity in the drug-treated cells. P-values were calculated using two-sided Welch's T-tests on the averages of replicates.

E) [^{18}F]FDG uptake by the cells used in A) following treatment with drug vehicle or GDC-0032 for 72 h. Figure shows the % of control cell [^{18}F]-FDG uptake in the drug-treated cells ± SD for each condition (n=3 to 6 experiments). P-values were calculated using two-sided Welch's T-tests.

F) Expression of *PTEN* determined by qPCR and normalized by the geomean of *β-ACTIN* and *B2M* in GDC-0032 sensitive (Ctrl; PTEN wt) and PTEN-knockdown drug-resistant tumors (PTEN KD) recovered at the end of the imaging protocol. Figure shows the mean ± SD per condition (triplicates). P-values were calculated using two-sided Welch's T-tests.

G) Volumes (cm^3) of T47D PTEN wt (Ctrl), PTEN-knockdown (PTEN KD) and PTEN-knockout (PTEN KO) tumors were determined using caliper measurements before (pre) and after 3 doses of GDC-0032 or drug vehicle. For vehicle-treated tumors, n=10 for Ctrl, n=3 for PTEN KD and n=6 for PTEN KO and for GDC-0032-treated tumors n=7 for Ctrl, n=4 for PTEN KD and n=7 for PTEN KO. P-values were calculated using two-sided Welch's T-tests.

H) Western blots of P-Akt in T47D PTEN wt (Ctrl), or PTEN-knockout (PTEN KO) tumors recovered at the end of treatment with 3 doses of GDC-0032 or drug vehicle.

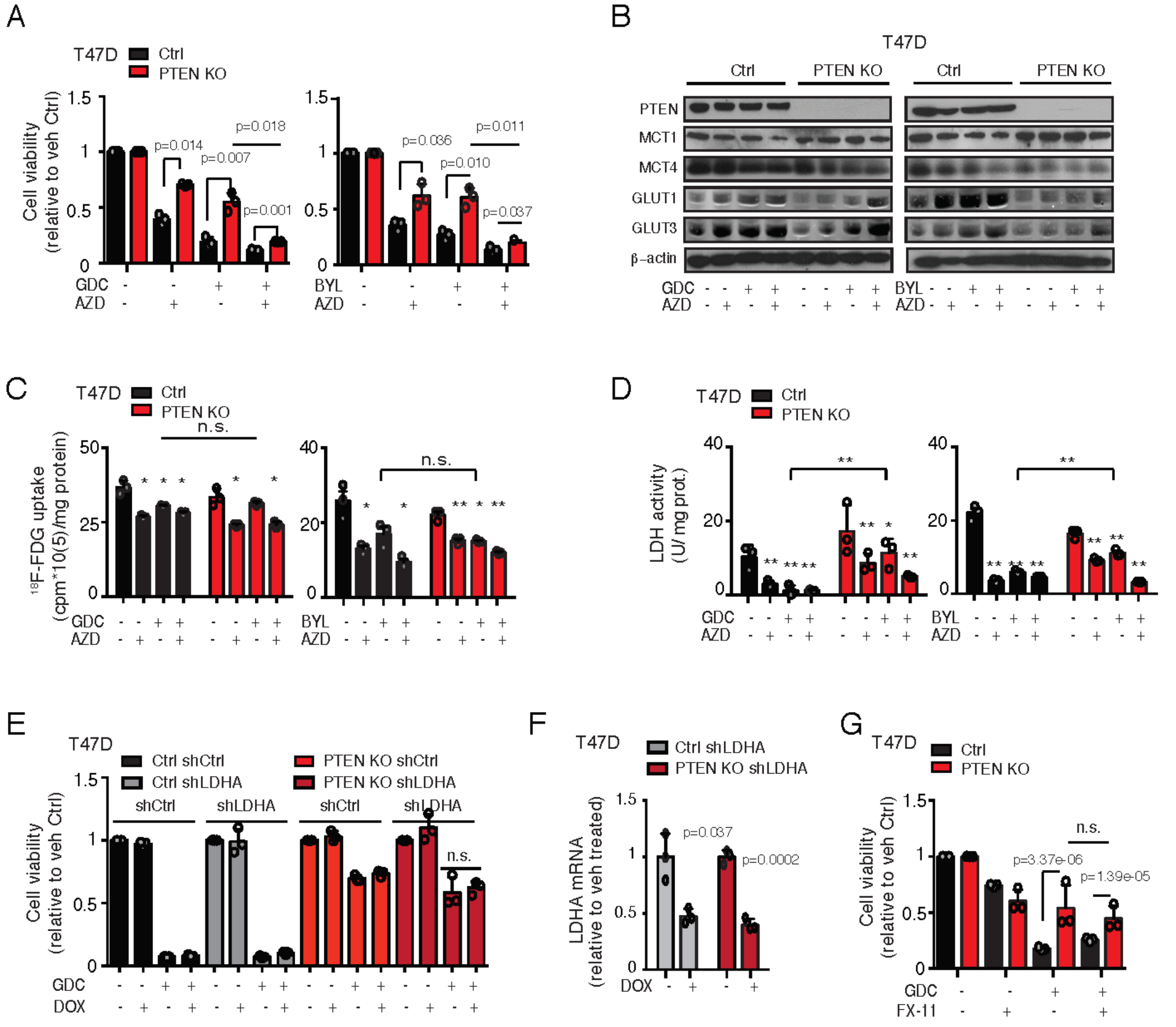

Figure S3, related to Figure 3. Combination treatments that overcome resistance to PI3Kα-specific inhibition result in decreased LDHA expression

A) Viabilities of T47D PTEN wt (Ctrl) and PTEN-knockout (PTEN KO) cells treated with the indicated drug combinations for 120 h, starting 24 h after plating. Figure shows the mean ± SD per condition (n=3, 5 technical replicates). P-values were calculated using two-sided Welch’s T-tests on the averages of technical replicates.

B) [^{18}F]FDG uptake in PTEN wt (Ctrl) and PTEN KO T47D cells treated with the indicated drug combinations for 72 h. Figure shows the mean ± SD per condition (n=3). P-values were calculated using two-sided Welch’s T-tests on technical replicates, * p ≤ 0.05 and ** p ≤ 0.005.

C) LDH activity in PTEN wt (Ctrl) and PTEN KO T47D cells treated with the indicated drug combinations for 72 h. Figure shows the mean ± SD per condition (n=3). P-

values were calculated using two-sided Welch's T-tests on technical replicates, * $p \leq 0.05$ and ** $p \leq 0.005$.

D) Expression of PTEN, HK-II, LDHA, monocarboxylate (MCT1 and MCT4) and glucose transporters (GLUT1 and GLUT3) in PTEN wt (Ctrl) and PTEN KO T47D cells treated with the indicated drug combinations for 72 h.

E) Viabilities of PTEN wt (Ctrl) and PTEN KO T47D cells expressing doxycycline-inducible shRNA sequences targeting LDHA (shLDHA) or control sequences (shCtrl) and treated with the indicated drug combinations for 120 h. Figure shows the mean ± SD per condition (n=3, 5 technical replicates). P- values were calculated using two-sided Welch's T-tests on the averages of technical replicates.

F) Expression of *LDHA* in the cells used in (D) was determined by qPCR and normalized by the geomean of *β-ACTIN* and *B2M*. Figure shows the mean ± SD per condition (n=3). P values were calculated using two-sided Welch's T-tests on the averages of technical replicates.

G) Viabilities of PTEN wt (Ctrl) and PTEN KO T47D cells treated with the indicated drug combinations for 120 h. Figure shows the mean ± SD per condition (n=3, 5 technical replicates). P-values were calculated using two-sided Welch's T-tests on the averages of technical replicates.

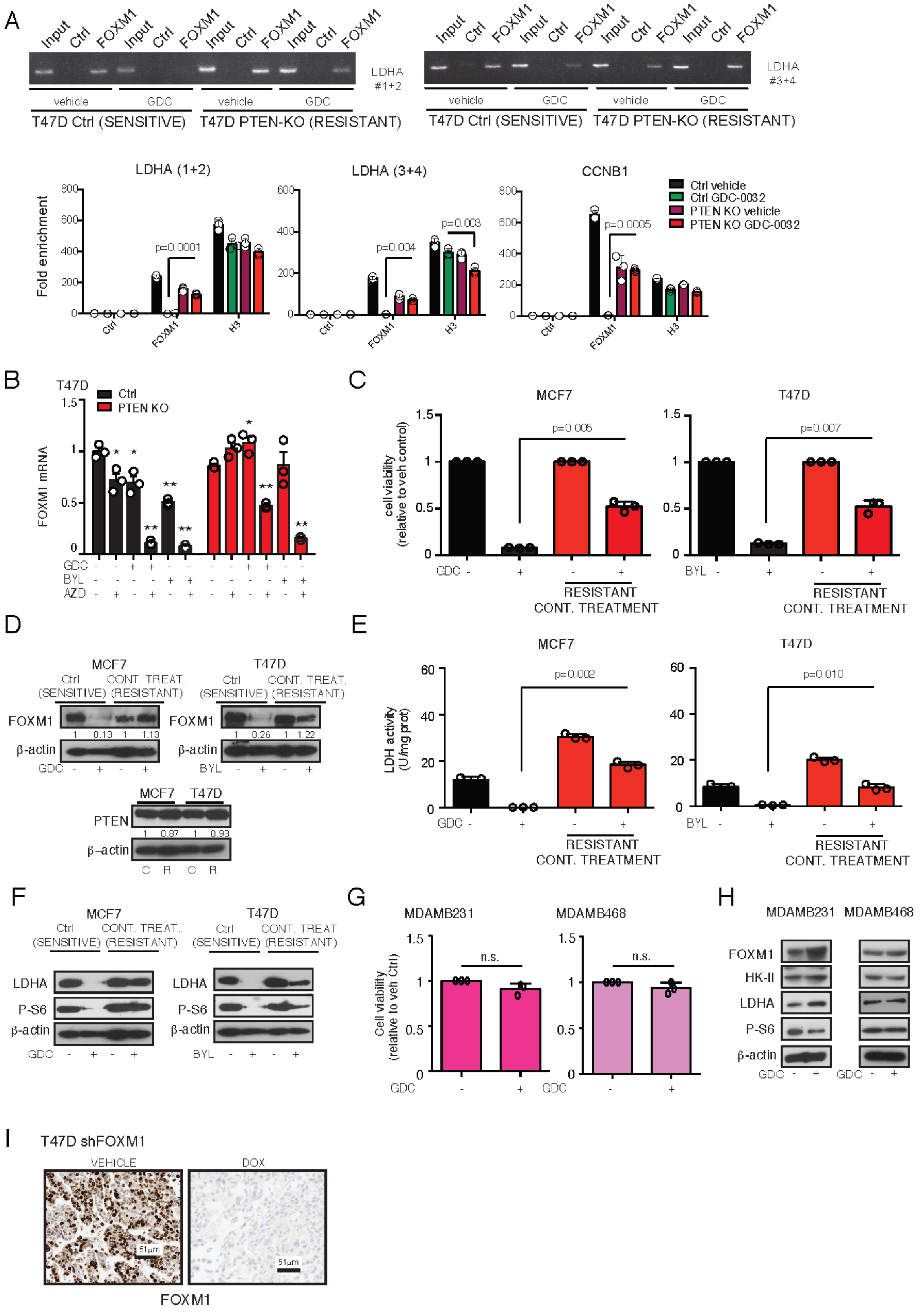
A
Input Ctrl FOXM1 Input Ctrl FOXM1 Input Ctrl FOXM1 Input Ctrl FOXM1
LDHA #1+2
vehicle GDC vehicle GDC
T47D Ctrl (SENSITIVE) T47D PTEN-KO (RESISTANT)
LDHA #3+4
LDHA (1+2)
LDHA (3+4)
CCNB1
Fold enrichment
p=0.0001
p=0.004
p=0.003
p=0.0005
Ctrl FOXM1 H3
Ctrl vehicle
Ctrl GDC-0032
PTEN KO vehicle
PTEN KO GDC-0032
B
T47D
Ctrl
PTEN KO
FOXM1 mRNA
GDC
BYL
AZD
C
MCF7
T47D
cell viability (relative to veh control)
p=0.005
p=0.007
RESISTANT CONT. TREATMENT
D
MCF7
T47D
Ctrl (SENSITIVE)
CONT. TREAT. (RESISTANT)
FOXM1
β-actin
1 0.13 1 1.13
1 0.26 1 1.22
PTEN
1 0.87 1 0.93
C R C R
E
LDH activity (U/mg prot)
p=0.002
p=0.010
F
LDHA
P-S6
β-actin
G
MDAMB231
MDAMB468
Cell viability (relative to veh Ctrl)
n.s.
H
FOXM1
HK-II
I
T47D shFOXM1
VEHICLE
DOX
51µm
FOXM1

Figure S4, related to Figure 4. PI3Kα inhibition decreases FOXM1 expression whereas drug-resistant tumors show sustained expression

A) T47D Ctrl and PTEN KO cells treated with vehicle or GDC-0032 for 72 h were subjected to chromatin immunoprecipitation assays using a FOXM1-specific antibody (FOXM1 Ab), a control antibody (IgG) and a positive control H3-antibody (H3 Ab). Promoter regions corresponding to 2 FOXM1-sites (oligos 1+2 or oligos3+4) in the *LDHA* promoter were amplified using qPCR, in three technical replicates. A FOXM1-site in the *CCNB1* promoter was used as a positive control. Figure shows the mean ± SD per condition (n=3). P-values were calculated using two-sided Welch's T-tests.

B) Expression of *FOXM1* in Ctrl (PTEN wt) and PTEN KO T47D cells treated with the indicated drug combinations for 120 h. FOXM1 mRNA levels were determined by qPCR and normalized by the geomean of *β-ACTIN* and *B2M*. Figure shows the mean ± SD per condition (n=3). P-values were calculated using two-sided Welch's T-tests on the averages of technical replicates, * $p \leq 0.05$ and ** $p \leq 0.005$.

C) Viabilities of MCF7, T47D, MCF7 cells rendered resistant to GDC-0032 and T47D cells rendered resistant to BYL-719 by prolonged drug treatment, following treatment for 120 h. Figure shows the mean ± SD per condition (n=3, 5 technical replicates). P-values were calculated using two-sided Welch's T-tests on the averages of technical replicates.

D) Upper panel; FOXM1 expression in PTEN wt (Ctrl) and PTEN KD MCF7 and T47D cells treated with indicated drug combinations for 72 h. Lower panel; Immunoblot of PTEN in MCF7 and T47D cells that had been rendered resistant to GDC-0032 or BYL-719 (R) respectively by prolonged exposure to the drugs compared to the parental lines (C) following treatment.

E) LDH activity in the cells used in C treated with GDC-0032 or BYL-719 for 96 h. Mean ± SD per condition (n=3). P-values were calculated using two-sided Welch's T-tests.

F) Expression of P-S6 and LDHA in the cells used in C treated with GDC-0032, or BYL-719 for 96 h. P values were calculated using two-sided Welch's T-tests.

G) Viabilities of MDAMB231 and MDAMB468 cells treated with GDC-0032 for 120 h. Mean ± SD per condition (n=3, 5 technical replicates). P-values were calculated using one-sample student T-tests on average the of technical replicates.

H) Immunoblot of cell lysates used in G) treated with GDC-0032, or BYL-719 for 96 h. I) Immunohistochemical staining for human FOXM1 expression in formalin-fixed paraffin-embedded sections of pellets of T47D cells that had been transduced with a lentiviral vector expressing a doxycycline-inducible shRNA against human FOXM1, after treatment with drug vehicle (left) or doxycycline (right) for 120 h.

A

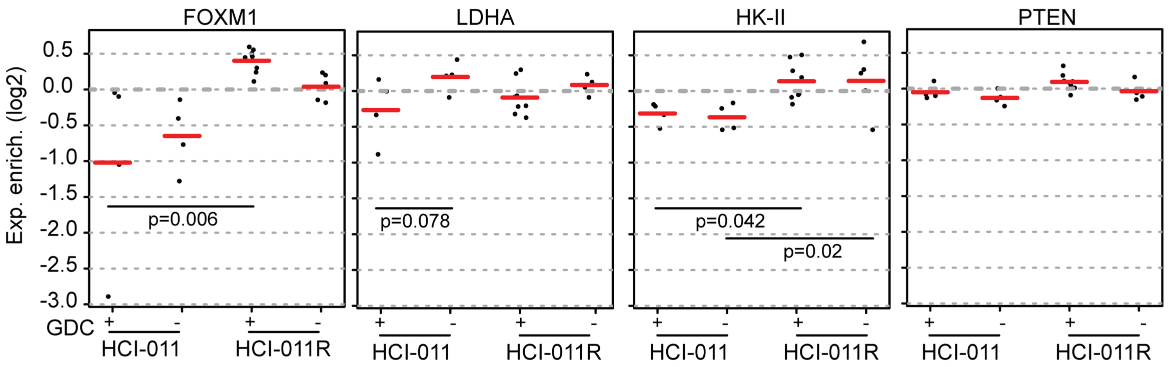

B

PATHWAY	N Genes	Direction	pValue	FDR
HALLMARK_G2M_CHECKPOINT	198	Up	9.64E-20	4.82E-18
HALLMARK_E2F_TARGETS	200	Up	4.00E-18	1.00E-16
HALLMARK_MITOTIC_SPINDLE	191	Up	7.47E-14	1.24E-12
HALLMARK_OXIDATIVE_PHOSPHORYLATION	197	Down	8.47E-04	8.61E-03
HALLMARK_PROTEIN_SECRETION	93	Up	8.61E-04	8.61E-03
HALLMARK_UV_RESPONSE_DN	123	Up	1.21E-03	1.01E-02
HALLMARK_TNFA_SIGNALING_VIA_NFKB	167	Down	1.43E-03	1.02E-02
HALLMARK_MTORC1_SIGNALING	195	Up	2.13E-03	1.33E-02
HALLMARK_SPERMATOGENESIS	73	Up	3.87E-03	2.15E-02
HALLMARK_INTERFERON_ALPHA_RESPONSE	89	Down	7.49E-03	3.47E-02
HALLMARK_INTERFERON_GAMMA_RESPONSE	155	Down	1.05E-02	4.48E-02
HALLMARK_ANDROGEN_RESPONSE	92	Up	1.07E-02	4.48E-02

C

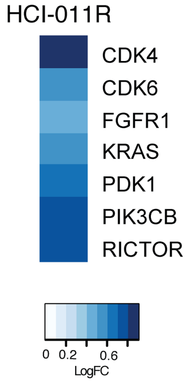

Figure S5, related to Figure 6: Persistent FOXM1 expression in breast cancer PDXs that have acquired resistance to PI3Kα inhibition

A) RNAseq analysis of *FOXM1*, *HK-II*, *LDHA* and *PTEN* gene expression in GDC-0032-treated drug-sensitive PDX model (HCI-011, vehicle n=4 and GDC-0032 n=4) and in the drug-resistant model derived from a tumor that relapsed following prolonged drug treatment (HCI-011R, vehicle n=5 and GDC-0032 n=8). Mean values are marked by red lines. FDR adjusted p-values of significant differentially expressed pairs are shown.

B) RNAseq data was used to perform a gene set enrichment analysis (GSEA) of hallmark pathways in HCI-011 (vehicle n=4 and GDC-0032 n=4) versus HCI-011R (vehicle n=5 and GDC-0032 n=8) tumors treated with GDC-0032, as in (A).

C) Heat map derived from analysis of the RNAseq data for HCI-011 vs HCI-011R PDX models for the genes listed as driving resistance to PI3K inhibition (Hanker et al., 2019), p value is < 0.01 or less.

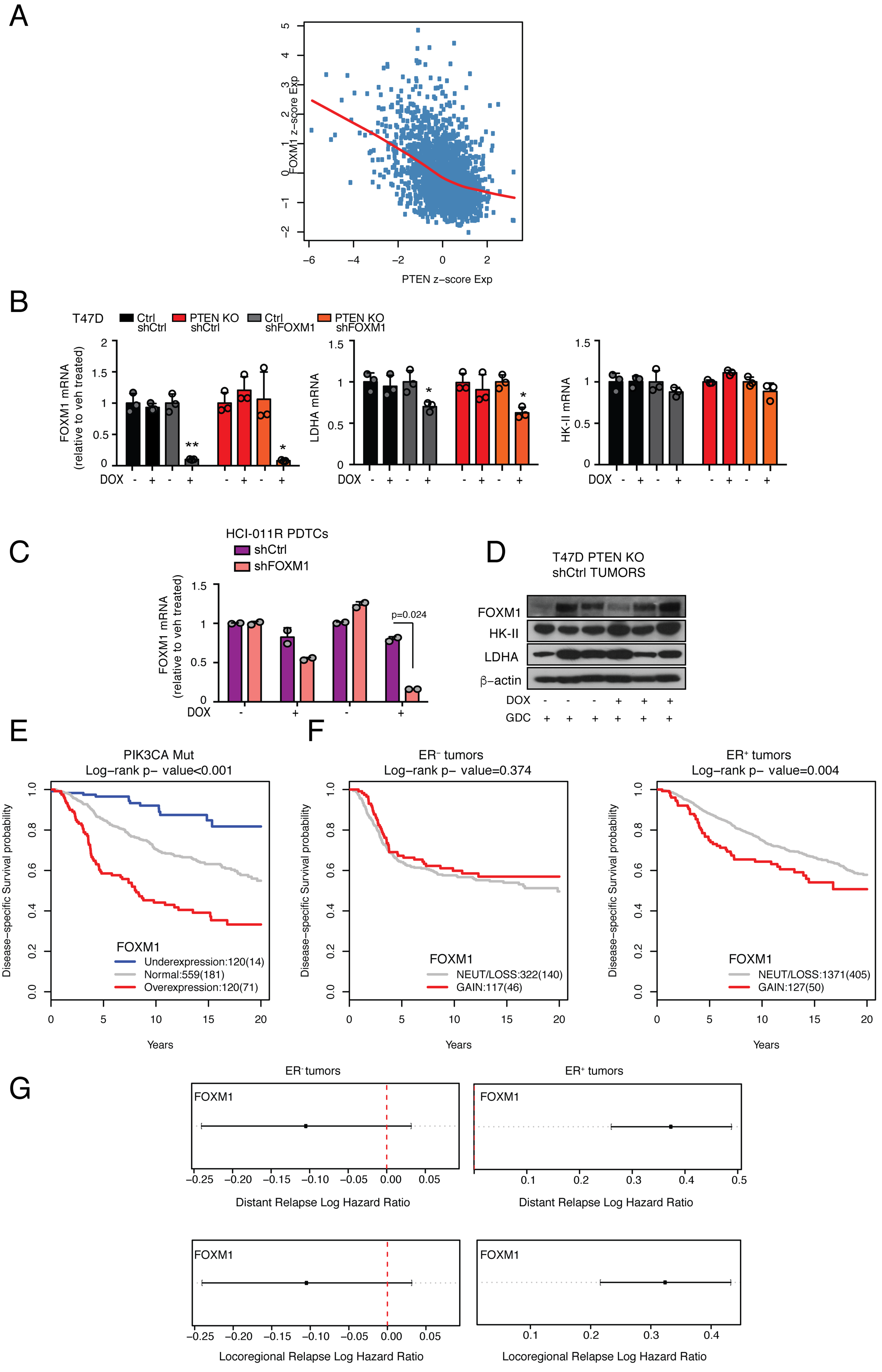
A
FOXM1 z-score Exp
PTEN z-score Exp
B
T47D
Ctrl shCtrl
PTEN KO shCtrl
Ctrl shFOXM1
PTEN KO shFOXM1
FOXM1 mRNA (relative to veh treated)
LDHA mRNA
HK-II mRNA
DOX
C
HCI-011R PDTCs
shCtrl
shFOXM1
FOXM1 mRNA (relative to veh treated)
p=0.024
DOX
D
T47D PTEN KO shCtrl TUMORS
FOXM1
HK-II
LDHA
β-actin
DOX
GDC
E
PIK3CA Mut
Log-rank p- value<0.001
Disease-specific Survival probability
FOXM1
Underexpression:120(14)
Normal:559(181)
Overexpression:120(71)
Years
F
ER⁻ tumors
Log-rank p- value=0.374
FOXM1
NEUT/LOSS:322(140)
GAIN:117(46)
ER⁺ tumors
Log-rank p- value=0.004
FOXM1
NEUT/LOSS:1371(405)
GAIN:127(50)
G
ER⁻ tumors
ER⁺ tumors
FOXM1
Distant Relapse Log Hazard Ratio
Locoregional Relapse Log Hazard Ratio

Figure S6, related to Figure 7: Persistent FOXM1 expression contributes to drug resistance and can be explained by cytoplasmic localization of FOXO3a

A) Pearson correlation of FOXM1 and PTEN expression in the METABRIC cohort (Curtis et al., 2012). A loess fit has been added to the plot.

B) Expression of *FOXM1, LDHA and HK-II* in PTEN wt (Ctrl) and PTEN KO T47D cells expressing a doxycycline-inducible shRNA sequence targeting FOXM1 (shFOXM1_seq1) treated with drug vehicle or doxycycline for 120 h determined by qPCR and normalized by the geomean of *β-ACTIN* and *B2M*. Figure shows the mean ± SD (n=3, 3 technical replicates). P-values were calculated using two-sided Welch's T-tests on the average of technical replicates, * $p \leq 0.05$ and ** $p \leq 0.005$.

C) Expression of *FOXM1* in HCI-011R PDTCs treated with indicated drug combinations for 120 h determined by qPCR and normalized by the geomean of *β-ACTIN* and *B2M*. Mean ± SD per condition (n=2). P-values were calculated using two-sided Welch's T-tests.

D) Immunoblot of FOXM1, HK-II and LDHA in PTEN KO T47D tumors expressing doxycycline-inducible shRNA control sequences (shCtrl) from mice treated with GDC-0032 (long-term treatment) and fed with doxycyline or a standard diet.

E) Disease specific Kaplan-Meier survival plots of breast cancer patients from METABRIC with low, medium and high FOXM1 expression (blue, grey and red lines, respectively). Under and over expression were defined by the 15% and the 85% percentiles of the distribution of expression respectively.

F) Disease specific Kaplan-Meier survival plots of ER^- and ER^+ breast cancer patients from METABRIC with FOXM1 copy number gain (red) or neutral/loss (grey).

G) Relapse Log-hazard ratio for FOXM1 expression in a multiple Cox regression model, for ER+ and ER- breast cancer patients from METABRIC.
